# Acquired deficiency of peroxisomal dicarboxylic acid catabolism is a metabolic vulnerability in hepatoblastoma

**DOI:** 10.1016/j.jbc.2021.100283

**Published:** 2021-01-13

**Authors:** Huabo Wang, Jie Lu, Xiaoguang Chen, Marie Schwalbe, Joanna E. Gorka, Jordan A. Mandel, Jinglin Wang, Eric S. Goetzman, Sarangarajan Ranganathan, Steven F. Dobrowolski, Edward V. Prochownik

**Affiliations:** 1 Division of Hematology/Oncology, Department of Pediatrics UPMC Children’s Hospital of Pittsburgh, Pittsburgh, Pennsylvania, USA; 2 School of Animal Science and Technology, Henan University of Science and Technology, Luoyang, Henan, People’s Republic of China; 3 Central South University Xiangya School of Medicine, Changsha, Hunan, People’s Republic of China; 4 Division of Medical Genetics, Department of Pediatrics, UPMC Children’s Hospital of Pittsburgh, Pittsburgh, Pennsylvania, USA; 5 Department of Pathology, Cincinnati Children’s Hospital, Cincinnati, Ohio, USA; 6 The Hillman Cancer Center, The University of Pittsburgh Medical Center, Pittsburgh, Pennsylvania, USA; 7 The Pittsburgh Liver Research Institute, Pittsburgh, Pennsylvania, USA; 8 The Department of Microbiology and Molecular Genetics, The University of Pittsburgh Medical Center, Pittsburgh, Pennsylvania, USA

**Keywords:** cancer metabolism, Ehhadh, fatty acid oxidation, hepatocellular carcinoma, metabolic reprogramming, oxidative phosphorylation, Warburg effect, peroxisome, AcCoA, acetyl coenzyme A, Cpt1a, carnitine palmitoyltransferase 1A, DDDA, dodecanedioic acid, ECM, extracellular matrix, Ehhadh, enoyl-CoA hydratase/3-hydroxyacyl CoA dehydrogenase, FAO, fatty acid oxidation, FDR, false discovery rate, HB, hepatoblastoma, HCC, hepatocellular carcinoma, HFDs, high-fat diets, OCRs, oxygen consumption rates, TCA, tricarboxylic acid, YAP, yes-associated protein

## Abstract

Metabolic reprogramming provides transformed cells with proliferative and/or survival advantages. Capitalizing on this therapeutically, however, has been only moderately successful because of the relatively small magnitude of these differences and because cancers may further adapt their metabolism to evade metabolic pathway inhibition. Mice lacking the peroxisomal bifunctional enzyme enoyl-CoA hydratase/3-hydroxyacyl CoA dehydrogenase (Ehhadh) and supplemented with the 12-carbon fatty acid lauric acid (C12) accumulate the toxic metabolite dodecanedioic acid (DDDA), which causes acute hepatocyte necrosis and liver failure. We noted that, in a murine model of pediatric hepatoblastoma (HB) and in primary human HBs, downregulation of Ehhadh occurs in association with the suppression of mitochondrial β- and endosomal/peroxisomal ω-fatty acid oxidation pathways. This suggested that HBs might be more susceptible than normal liver tissue to C12 dietary intervention. Indeed, HB-bearing mice provided with C12- and/or DDDA-supplemented diets survived significantly longer than those on standard diets. In addition, larger tumors developed massive necrosis following short-term DDDA administration. In some HBs, the eventual development of DDDA resistance was associated with 129 transcript differences, ∼90% of which were downregulated, and approximately two-thirds of which correlated with survival in numerous human cancers. These transcripts often encoded extracellular matrix components, suggesting that DDDA resistance arises from reduced Ehhadh uptake. Lower Ehhadh expression was also noted in murine hepatocellular carcinomas and in subsets of certain human cancers, supporting the likely generality of these results. Our results demonstrate the feasibility of C12 or DDDA dietary supplementation that is nontoxic, inexpensive, and likely compatible with more standard chemotherapies.

Cancer cells undergo numerous metabolic adjustments to acquire and maintain proliferative and survival advantages, and these represent essential features of the transformed state (1, 2, 3, 4, 5, 6, 7). The classic example of metabolic reprogramming is the Warburg effect, whereby the anerobic process of glycolysis continues or is even accelerated under aerobic conditions (7, 8, 9). The abundance of glycolytic intermediates provided by Warburg-type respiration is believed to serve as a ready source of the reducing equivalents, ribose sugars, nucleotides, and amino acids needed to sustain tumor growth. ATP production is also increased and may match or even exceed the levels attained by oxidative phosphorylation, the downregulation of which is a prominent feature of many cancers (8, 10, 11). Yet despite its diminished prominence, mitochondrial metabolism remains an important source of tricarboxylic acid (TCA) cycle–derived anabolic precursors, such as acetyl coenzyme A (AcCoA), *α*-ketoglutarate, succinate, and oxaloacetate (1, 12, 13). Indeed, the upregulation of pathways that provide anaplerotic sources of TCA cycle substrates is another important metabolic feature of many cancers (1, 2, 5, 13, 14, 15). Many studies have attempted, with variable success, to leverage some of the more prominent metabolic disparities between normal and transformed cells for therapeutic benefit (1, 4, 9, 14, 16, 17).

We previously observed that murine models of pediatric and adult liver cancer, namely hepatoblastoma (HB) and hepatocellular carcinoma (HCC), as well as their human counterparts, also remodel their metabolism and become more reliant on glycolysis than on fatty acid oxidation (FAO). Consistent with this, total mitochondrial mass and transcripts encoding virtually all enzymes in the mitochondrial-based β-FAO pathway are markedly downregulated (18, 19, 20, 21, 22).

In addition to β-FAO, most cells also engage in the nonenergy-generating ω-oxidation of very long–chain fatty acids >20 carbons in length. However, an additional role of this pathway is to catabolize a significant fraction of lauric acid, the 12-carbon medium-chain fatty acid that is too small to be efficiently transported by the fatty acid carrier carnitine palmitoyltransferase 1A (Cpt1a) and too large to enter mitochondria passively as do short-chain fatty acids. ω-oxidation initiates in the endoplasmic reticulum *via* reactions involving the cytochrome P450 members Cyp4a10 (CYP4a11 in humans) and Cyp4a14, with subsequent catabolism occurring *via* peroxisomal β-oxidation. Some of the end products of this pathway such as succinate and AcCoA can be utilized for anabolic purposes, whereas others, such as adipate and suberate, are excreted.

Lauric acid (hereafter C12) is the main fatty acid component of coconut oil (23) and is metabolized by hepatocytes to dodecanedioic acid (DDDA), a highly toxic dicarboxylic acid that is further metabolized by the bifunctional enzyme enoyl-CoA hydratase/3-hydroxyacyl CoA dehydrogenase (Ehhadh) (24). In contrast to normal mice, which readily tolerate either C12- or DDDA-enriched diets, *ehhadh−/−* mice accumulate high levels of DDDA, develop hepatic inflammation and necrosis, and succumb within days to acute liver failure (25, 26).

In addition to downregulating mitochondrial β-oxidation, we show here that experimental murine HBs and HCCs also markedly suppress the ω-oxidation/peroxisomal pathway (18, 20, 21). This suggested that these tumors and perhaps other cancers possess a metabolic vulnerability in the form of C12 and/or DDDA intolerance analogous to that of *ehhadh−/−* mice. Indeed, we also show that the life spans of HB-bearing mice can be significantly extended by dietary supplementation with C12 and/or DDDA provided shortly after tumor initiation. Similarly, large pre-existing tumors develop massive necrosis in response to short-term implementation of DDDA-enriched diets. Several different human cancer types, including HB and HCC, also show striking downregulation of *ehhadh* expression suggesting that these too might be sensitive to C12 and/or DDDA. Collectively, our studies identify a novel metabolic susceptibility that could be targeted *via* relatively simple means and with minimal toxicity without compromising standard chemotherapeutic options.

## Results

### C12- and/or DDDA-enriched diets extend the life span of HB-bearing mice

**Figure 1:**
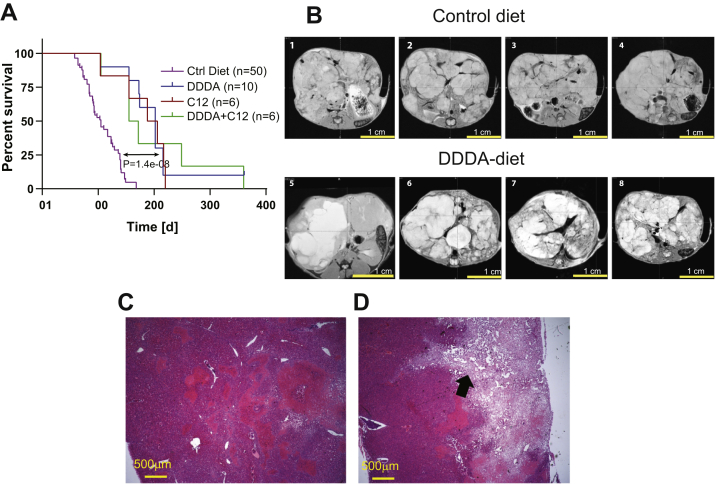
**C12 and DDDA diets impair long-term hepatoblastoma (HB) growth and induce massive necrosis.***A,* survival curves of HB-bearing mice. Each of the indicated cohorts was injected with Sleeping Beauty vectors encoding Δ(90) and YAP^S127A^ (19, 20, 21, 22, 27). One week later, the indicated diets were initiated for the remainder of the study. Kaplan–Meier survivals for each group were determined by the log-rank test. *B*, tumors were generated as described in *A* except that mice were maintained on standard diets for 8 weeks. Half of the mice were then provided DDDA diets, and the remaining mice were maintained on standard diets. MRIs were performed on each group at week 11. Representative axial sections from four mice in each group are shown. *C,* low-power magnification (5×) of a typical H&E-stained section from a standard diet HB obtained at the time of sacrifice. *D,* similar H&E section of a typical HB from a mouse maintained for 3 weeks on a DDDA diet as described in *B.* A large area of necrosis extending from the tumor surface into the parenchyma is indicated by the *arrow*. DDDA, dodecanedioic acid.

HBs were generated by hydrodynamic tail vein injection of Sleeping Beauty vectors encoding a patient-derived 90 bp in-frame oncogenic deletion mutant of β-catenin (hereafter, Δ(90)) and a missense mutant (S127A) of yes-associated protein (hereafter, YAP^S127A^), the terminal effector of the Hippo tumor suppressor pathway (19, 20, 21, 22, 27). We refer to these HBs hereafter as Δ(90) tumors. After allowing 4 weeks for tumor initiation, the animals were divided into four dietary cohorts: (1) standard (control) diet, (2) standard diet + 10% (w/w) C12 in the form of glycerol trilaurate (C12 diet), (3) standard diet + 10% (w/w) DDDA (DDDA diet), and (4) standard diet + C12 and DDDA (10% w/w each) (C12 + DDDA diet). As previously reported, median survival in the former group was 14.2 weeks (19, 20, 21, 22, 27) (Fig. 1*A*). In contrast, survival among each of the other groups was significantly longer although they were similar among themselves (range, 22.8–28.6 weeks). These results show that two structurally distinct substrates of the ω-oxidation/peroxisomal pathway, both individually and together, significantly extended survival in this model of aggressive HB, which mimics the β-catenin and YAP dysregulation commonly encountered in human tumors (27, 28, 29, 30).

To determine whether the aforementioned interventions impacted the growth of larger HBs that better reflect human disease, we generated additional Δ(90) tumors, initiated DDDA diets in half the animals after tumors had attained a moderately large size at 8 weeks, and then evaluated the tumors 3 weeks later by MRI. As expected, mice maintained on standard diets had massive HBs that displaced adjacent organs. They also succumbed by week 12 to 13 with tumors of 10 to 14 g (Fig. 1*B*) (19, 20, 22). In contrast, tumors from mice maintained on DDDA diets showed areas of extensive necrosis that were confirmed by histologic examination of H&E-stained tumor sections (Fig. 1, *B*–*D*). Control HBs contained densely packed regions of small and round blue cells with the crowded fetal histology that typifies Δ(90) tumors and represents the most common HB subtype (19, 20, 21, 22, 27, 29, 30). While histologically similar, DDDA diet tumors contained large areas of consolidative necrosis. Together with the previous results, these findings indicate that C12 and/or DDDA slow HB progression and promote regression by inducing extensive tumor cell death.

### Tumors downregulate mitochondrial and peroxisomal FAO

**Figure 2:**
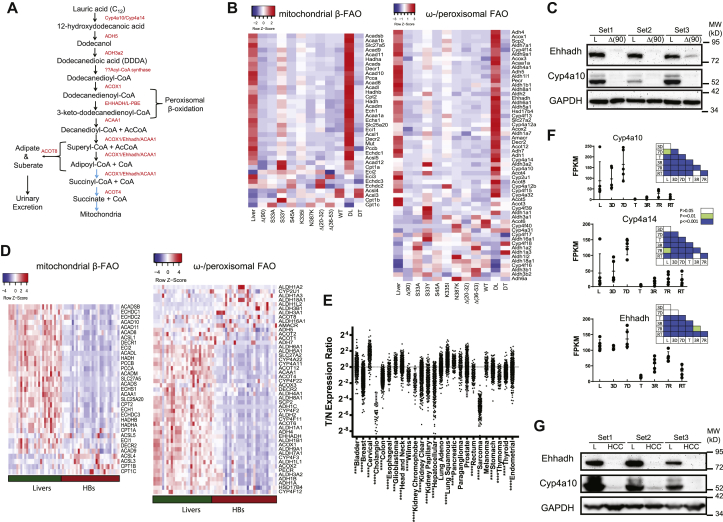
**Parallel downregulation of the β-fatty acid oxidation (β-FAO) and ω-/peroxisomal-FAO pathways in murine and human cancers.***A,* the pathway of C12 metabolism. ω-oxidation initiates in endosomes and is catalyzed by Cyp4a10 and Cyp4a14. The resulting dicarboxylic acids, including DDDA, are further catabolized in this compartment and the peroxisome. The ultimate products, adipate and suberate, are excreted in urine, whereas succinyl-CoA and succinate supply the tricarboxylic acid cycle or other biosynthetic pathways. *B*, RNA-Seq results. Heat maps showing transcript levels for mitochondrial β-FAO and ω-/peroxisomal-FAO pathways in livers and Δ(90) hepatoblastomas (HBs) maintained on standard and DDDA diets for 3 weeks. DL and DT = livers and tumors, respectively, from DDDA-diet cohorts. In addition, transcripts are shown from standard diet–maintained tumors generated by other previously described patient-derived β-catenin mutants (22). *C,* enoyl-CoA hydratase/3-hydroxyacyl CoA dehydrogenase (Ehhadh) and Cy4a10 protein expression from three independent sets of livers and Δ(90) β-catenin mutant–generated HBs, all from mice maintained on standard diets. *D,* heat maps as described in *B* generated from the RNA-Seq results of 25 human HBs (32). *E*, expression of Ehhadh transcripts from the indicated human tumors (T) relative to that of matched and normal control tissues (N) from The Cancer Genome Atlas (TCGA) RNA-Seq data. *F,* absolute expression (FPKM) of transcripts encoding Cy4a10, Cyp4a14, and Ehhadh in tissues from mice bearing a doxycycline-responsive human Myc transgene. Results are taken from RNA-Seq data published by Dolezal *et al*. (18). L: control (Myc-suppressed) livers prior to Myc induction; 3 and 7 days: livers without obvious tumors 3 and 7 days after withdrawal of doxycycline and the induction of Myc; T: tumor samples approximately 4 weeks after the induction of Myc; 3R and 7R: regressing tumors 3 and 7 days following Myc silencing by doxycycline reinstatement. RT: recurrent tumor. Initial tumors were allowed to regress for approximately 3 months at which point no residual tumor could be detected (18, 33). Recurrent tumors were then induced by the removal of doxycycline and sampled after 3 to 4 weeks of tumor regrowth. Insets: *p* values between groups show on the abscissas and ordinates. *G,* expression of Ehhadh and Cyp4a10 in three sets of control livers and Myc-driven initial HCCs from F. FPKM, fragments per kilobase million.

Δ(90) HBs coordinately downregulate mitochondrial mass by ∼80% as they switch their primary mode of energy generation from mitochondrial β-FAO to glycolysis (19, 20, 21, 22). We confirmed this but also observed a similar downregulation of transcripts for endosomal ω- and peroxisomal FAO (hereafter, ω-/peroxisomal FAO), which is responsible for the metabolism of very long chain fatty acids and C12 (24, 25, 31); as a group, they were 3.1-fold downregulated (*p* = 2.6 × 10^−12^; Fig. 2, *A* and *B*). These transcripts included those for Ehhadh (7.7-fold downregulated, *p* = 1.11 × 10^−16^) and the microsomal P450 cytochromes, Cyp4a10 and Cyp4a14, which catalyze the first step in the catabolism of C12 (13.1-fold and 35.8-fold downregulated, respectively, *p* = 4.31 × 10^−7^ and *p* = 6.98 × 10^−11^) (34). This paralleled an equally marked decline at the protein level (Fig. 2*C*).

In concert with YAP^S127A^, numerous other patient-derived β-catenin mutants are tumorigenic in mice (22). These grow at different rates and display diverse histologic subtypes, which in some cases more closely resemble HCCs than HBs. However, like Δ(90) tumors, they all broadly downregulated β-FAO and ω-/peroxisomal FAO enzyme transcripts (Fig. 2*B*; (22)). The suppression of the latter pathway is thus widespread, occurs in parallel with mitochondrial β-FAO–related transcript downregulation, and is independent of tumor growth rate. The RNA-Seq transcriptional profiles of 25 previously reported primary human HBs (32) showed that, like murine HBs, these tumors also downregulated both β-FAO and ω-/peroxisomal pathways (Fig. 2*D*).

Among the 33 cancers in The Cancer Genome Atlas for which a sufficient number of samples plus matched control normal tissues were available, we identified at least six tumor types where ω-/peroxisomal-FAO transcripts as a group were significantly downregulated, although considerable variability was seen among individual tumors (Fig. S1). Those containing subsets with particularly low expression of Ehhadh included HCCs, cholangiocarcinomas, kidney cancers, and sarcomas (Fig. 2*E*).

Poorly differentiated aggressive HCCs arise in mice in response to the conditional overexpression of c-Myc (Myc) (18, 33). These tumors regress rapidly and completely following Myc silencing and recur following its re-expression. We followed Cyp4a10, Cyp4a14, and Ehhadh transcripts across this time course using our previously obtained RNA-Seq data (18). In contrast to Ehhadh transcripts, which remained unchanged during the earliest stages of tumor induction and prior to their actual appearance, Cyp4a10 and Cyp4a14 transcripts were initially induced in a time-dependent manner (Fig. 2*F*). However, all three transcripts coordinately fell to near undetectable levels in the tumors that subsequently appeared, thus mimicking their behavior in HBs (Fig. 2*B*). A partial normalization of transcripts, particularly those encoding Ehhadh, occurred during regression. Recurrent tumors, like the initial ones, also expressed low levels of all three transcripts. Finally, as documented with HBs, Ehhadh and Cyp4A10 protein levels were virtually undetectable in tumors (Fig. 2*G*).

Finally, we evaluated Ehhadh protein expression in select normal adult human tissues and cancers from the Human Protein Atlas (https://www.proteinatlas.org/ENSG00000113790-EHHADH/pathology/) that were chosen based on the transcript expression results from Figure 2*E* and Figure S1. Very low-absent staining was seen in a number of cholangiocarcinomas, HCCs, and kidney cancers relative to their normal tissues (Fig. S2*A*). We also examined a tissue microarray containing 40 primary HBs and found 25 of these (62.5%) to express low-undetectable levels of Ehhadh (Fig. S2*B*). In tumor areas where staining was patchy or of low intensity, it tended to be more associated with fetal-like HB morphology, whereas areas with embryonal and/or pleomorphic cellular content were always negative. Collectively, these results showed that the coordinate downregulation of the mitochondrial β-FAO and ω-/peroxisomal-FAO pathways occurs in both murine HBs and HCCs, that a similar downregulation occurs in other cancers, and that ω-/peroxisomal-FAO pathway suppression is a frequent albeit variable finding across certain human cancers, particularly HBs.

### Low levels of β-FAO and ω-/peroxisomal-FAO transcripts are predictive of shorter survival in several cancer types.

**Figure 3:**
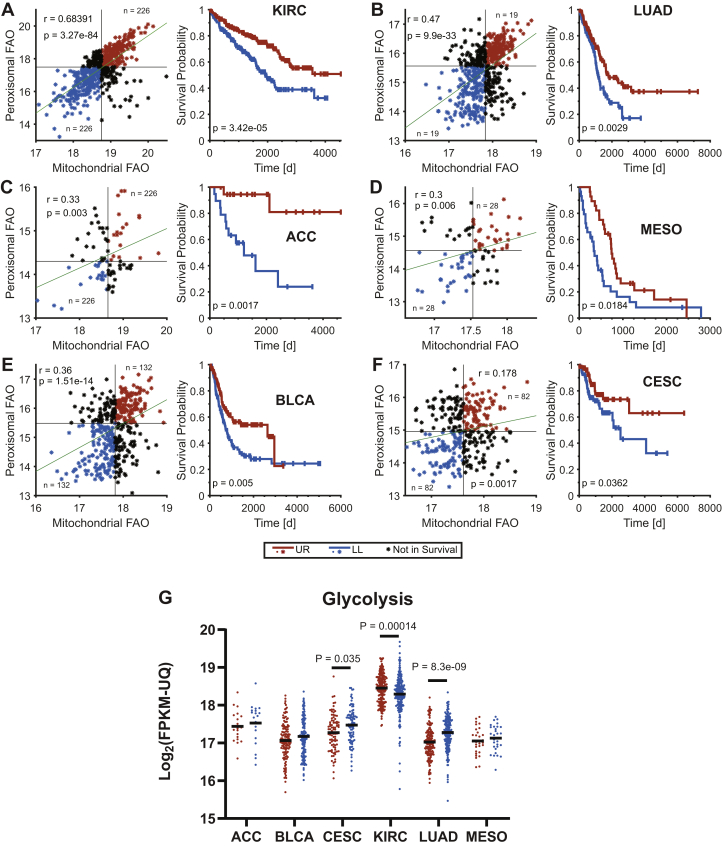
**Mitochondrial β-fatty acid oxidation (β-FAO)- and ω-/peroxisomal FAO-associated transcripts correlate directly with long-term survival in select cancers.** Transcripts used were those from Figure 2, *A* and *B* and Refs. (35) and (36)). FPKM-UQ values were obtained from the Genomic Data Commons-PANCAN dataset via the University of California Santa Cruz Xenabrowser (xena.ucsc.edu). Average expression for each set of transcripts was determined from all 33 cancers in The Cancer Genome Atlas (TCGA) and expressed as a dot plot, which was then divided into four quadrants, each containing approximately equal numbers of tumors. Long-term survival information was then obtained for the two quartile of patients whose tumors' transcript levels localized to the upper right and lower left portions of the dot plots. *A,* kidney renal clear cell carcinoma (KIRC). *B,* lung adenocarcinoma (LUAD). *C*, adrenocortical carcinoma (ACC). *D,* mesothelioma (MESO). *E,* bladder urothelial carcinoma (BLCA). *F,* cervical squamous cell carcinoma (CESC)/endocervical adenoma carcinoma. *G,* the average expression of transcripts encoding glycolytic enzymes (35, 36) was obtained from each of the aforementioned favorable and unfavorable survival cohorts. *p* values were determined using Welch's *t* test. FPKM-UQ, fragments per kilobase of transcript per million mapped reads upper quartile.

β-FAO- and ω-/peroxisomal-FAO–related transcript levels correlated in a number of human cancers and in at least six types, tumors with the lowest levels of both pathways' transcripts were associated with shorter median survival (Fig. 3, *A*–*F*). We previously noted an inverse correlation between mitochondrial β-FAO and glycolysis in HBs and HCCs and attributed this to the preservation of the Randle cycle, whereby these two competing metabolic pathways engage in a negative feedback loop in response to the activities of Cpt1a, phosphofructokinase, and the pyruvate dehydrogenase complex and their regulation by metabolites, such as ATP, AcCoA, malonyl CoA, and citrate (2, 18, 20, 21, 37, 38). In cervical squamous cell carcinoma and lung adenocarcinoma, tumors expressing the lowest β-FAO– and ω-/peroxisomal-FAO–related transcript levels tended to express higher levels of glycolysis-related transcripts, whereas clear cell kidney cancers associated with shorter survival expressed lower levels of glycolysis-related transcripts (Fig. 3*G*).

### The response to DDDA is associated with the induction of inflammatory markers and eventual DDDA resistance

**Figure 4:**
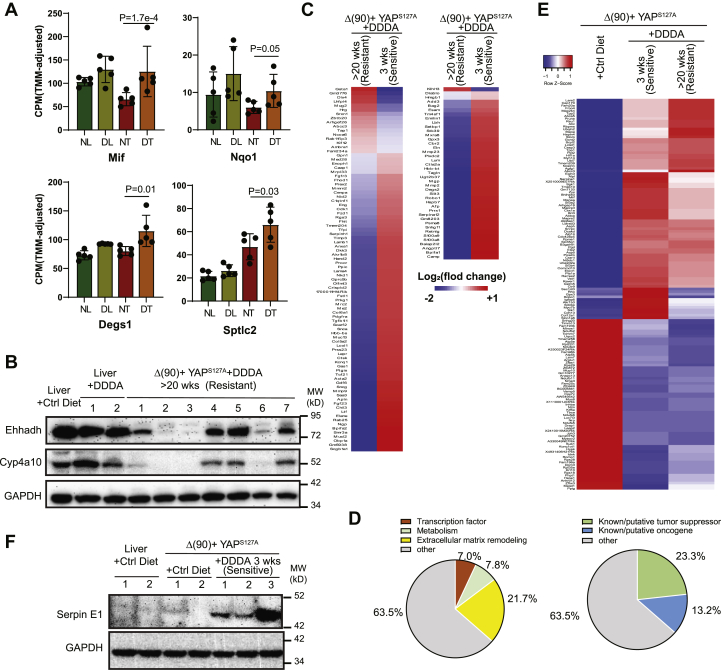
**Inflammatory mediators and properties of DDDA-diet–resistant tumors.***A,* levels of transcripts encoding the indicated inflammatory markers, phase II antioxidants, and sphingomyelin biosynthetic enzymes. Expression values (fragments per kilobase million) of the indicated transcripts were mined from RNA-Seq results of each cohort (N = 5/group). *B,* Ehhadh and Cyp4a10 protein expression in livers and tumors from mice maintained on standard diets and similar to those shown in Figure 2*C* were compared with those from mice maintained on DDDA diets for >20 weeks. Four sets of tissues from each group were analyzed. *C,* RNA-Seq analysis on tumor groups maintained for 3 weeks (Fig. 1*A*) or >20 weeks on DDDA-supplemented diets (N = 5/group). A total of 129 differences are shown here and includes both q < 0.05 and q > 0.05 but *p* < 0.001 groups. See Tables S1 and S2 for these gene sets. *D,* pie chart of the functional categories of transcripts depicted in *C* and Tables S1 and S2. *E,* RNA-Seq expression of the 149 differentially expressed transcripts in HBs maintained on standard diets *versus* DDDA diets for 3 weeks (N = 5/group). These transcripts comprised members of IPA pathways whose differences were the greatest between the tumor groups (Table S4). Also shown are the same transcripts from tumors that recurred following >20 weeks of DDDA-diet supplementation taken from panel C. *F,* expression of serpin E1 in the indicated tissues. DDDA, dodecanedioic acid; Ehhadh, enoyl-CoA hydratase/3-hydroxyacyl CoA dehydrogenase.

The rapid onset of acute hepatic failure in *ehhadh−/−* mice provided DDDA-containing diets is associated with the induction of select transcripts encoding inflammatory markers and enzymes that participate in phase II antioxidant response and sphingomyelin biosynthesis (25). We evaluated several of these in the livers and HBs of mice maintained on standard or DDDA diets for 3 weeks at which time RNA-Seq was performed from regions of tumors containing healthy-appearing tissue. In HBs from the DDDA diet cohort, we noted significant increases in transcripts encoding macrophage migration inhibitory factor (Mif), phase II enzyme NAD[P]H:quinone oxidoreductase 1 (Nqo1), and the sphingolipid pathway enzymes delta 4-desaturase Degs1 and serine palmitoyl-transferase 2 (Sptlc2) (Fig. 4*A*). Together with the results presented in Figure 1, *B* and *D*, these findings indicate that, in response to 3 weeks of dietary DDDA, HBs upregulate some of the same markers detected in the livers of C12 diet–maintained *ehhadh−/−* mice (25).

Despite objective responses to both long- and short-term C12 and/or DDDA diets (Fig. 1), HB-bearing mice eventually succumbed to progressive disease (Fig. 1*A*) with tumors no longer displaying the widespread necrosis that characterized the initial response (Fig. 1, *B* and *C*). Both Ehhadh and Cyp4a10 re-expression as a potential explanation for this acquired resistance were observed in some but not all tumors from mice maintained on DDDA diets for >20 weeks (Fig. 4*B*). We therefore performed another RNA-Seq analysis on tumors from the resistant cohort that maintained low levels of Ehhadh and Cyp4a10 expression and compared the results to the previously described HBs that initially responded to a 3-week DDDA diet with the intention of revealing the strategies employed by the former group to achieve ω-/peroxisomal-FAO pathway–independent DDDA resistance. To ensure inclusion of the broadest population of differentially expressed transcripts, we included those identified using any one of the three previously employed methods to quantify differential read counts, that is, edgeR, CLC Genomics Workbench, and DESeq2 (q < 0.05) and identified 41 differences. We then broadened the survey to include transcripts with q values >0.05 but *p* values <0.001 and identified an additional 88 differences (Fig. 4*C* and Tables S1 and S2).

About 112 of the aforementioned 129 transcripts (86.8%) were downregulated in the resistant cohort, with 23 of these (20.5%) being reduced >10-fold and only nine (8.0%) being reduced less than twofold. In contrast, none of the 17 upregulated transcripts were increased by >10-fold and eight (47.0%) were increased less than twofold. Thus, the resistant HB cohort was characterized by disproportionate and more robust transcript repression. About 28 (21.7%) mRNAs encoded structural components or remodelers of the extracellular matrix (ECM) or molecules functioning in cellular adhesion (Fig. 4*D*). Other prominent groups encoded transcription factors/coactivators (nine transcripts = 7.0%) and enzymes regulating metabolism or redox balance (10 transcripts = 7.8%). Perhaps most strikingly however, 30 (23.3%) transcripts encoded known or putative tumor suppressors, all but one of which were downregulated in resistant tumors. An additional 17 transcripts (13.2%) encoded known or putative oncoproteins or proteins that facilitate metastasis although only three of these were upregulated. Thus, in total, >70% of the differentially expressed transcripts between DDDA-sensitive and DDDA-resistant cohort encode proteins that likely alter tumor behavior, metabolism, and extracellular environment.

The transcripts listed in Tables S1 and S2 and their relationship to long-term survival were compiled from nearly 8000 samples of 17 different adult human cancers archived in The Human Protein Atlas (version 19.3) (https://www.proteinatlas.org/humanproteome/pathology). This showed that the expression of 63.6% of the transcripts (82 of 129) correlated with survival in one or more tumor types (Table S3). In 145 of the 181 matches identified (80.1%), the correlation with survival was concordant (*p* = 1.78 × 10^−7^, Pearson's Chi^2^ test). In addition, the tumor types in which these correlations were observed were not random. For example, the three most common kidney cancers (clear cell renal cancer, papillary renal cancer, and chromophobe renal cancer) and HCC (hepatocellular carcinoma) were found to have the most frequent associations (92 and 22 examples, respectively), whereas only a single association or none at all was seen for gliomas, melanomas, and cancers of the prostate and testes. These results indicate that DDDA-resistant HBs tended to downregulate transcripts associated with longer survival and upregulate transcripts associated with shorter survival and that these relationships were preserved in select subsets of tumors.

Finally, we asked if we could gain insight into the underlying mechanism(s) of tumor cell killing in response to short-term C12- or DDDA-supplemented diets. We therefore again exploited edgeR, CLC Genomics Workbench, and DESeq2 to identify differentially expressed transcripts in tumors from mice maintained on standard *versus* DDDA-enriched diets for 3 weeks. Depending on the analytic method used, the two tumor cohorts differed in the expression of 254 to 396 transcripts. Of these, 149 were identified by all three analytic methods (q < 0.05), with 84 being upregulated in the 3-week DDDA dietary group and 65 being downregulated (Fig. 4*E*). Functional categorization of these transcripts using Ingenuity Pathway Analysis software showed that at least 22 of these transcripts could be grouped into several major pathways that included mitochondrial, electron transport chain, and ribosomal/eukaryotic translation initiation factor 2 functions (Table S4). Additional upregulated transcripts encoded Caspase 3 and members of the nuclear factor erythroid 2–related factor 2 oxidative stress response pathway, including, serpinE1, Hif1α, and glutathione-*S*-transferase m5. Tumors from mice maintained on DDDA-supplemented diets for >20 weeks showed a pattern of expression more akin to that of the 3-week DDDA tumor group with the exception of a group of 12 transcripts that was uniformly highly downregulated so as to resemble the expression pattern of control livers (Fig. 4*E* and Table S5). At least eight of these (67%) encoded proteins with established roles in endothelial cell function and ECM environment and its remodeling. These findings suggested that the initial response to dietary DDDA involves altered regulation of oxidative phosphorylation and the electron transport chain, protein translation, and the oxidative stress response. With time, DDDA-resistant tumors in addition downregulate the expression of genes involved in cellular adhesion and ECM remodeling.

The second most highly upregulated transcript in 3-week DDDA tumors (8.2-fold induced relative to standard and control diet tumors and 60.4-fold induced relative to liver) was that encoding serpin E1, also known as plasminogen activator inhibitor 1, which, in addition to its canonical role in fibrinolysis, also participates in tumor growth, angiogenesis, metastasis, and ECM remodeling (39). We recently showed serpin E1 levels to be modestly elevated in HBs generated by Δ(90) + YAP^S127A^ but highly induced in response to concurrent nuclear factor erythroid 2–related factor 2 dysregulation. Moreover, the enforced hepatic coexpression of serpin E1 with Δ(90) + YAP^S127A^ + serpin E1, while not influencing tumor growth rates, was associated with widespread tumor parenchymal necrosis (https://www.biorxiv.org/content/10.1101/2020.09.13.295311v1). We confirmed the low-level upregulation of serpin E1 protein in standard diet–maintained HBs, and its marked induction in HBs maintained for 3 weeks on DDDA diets, during which time they developed the extensive necrosis depicted in Figure 1*B* (Fig. 4*F*).

### Effects of DDDA diets on liver and tumor metabolism

The normal liver is more reliant on β-FAO than glycolysis as its primary energy source, whereas the reverse is true for HBs and HCCs (18, 19, 22, 38). This is consistent with Warburg-type respiration but is not irreversible as it can be partially normalized by high-fat diets (HFDs), which restore liver-like levels of β-FAO, slow tumor growth, and extend survival (38). This “forced metabolic normalization” likely reflects the preservation of Randle cycle regulation discussed previously and the need for high rates of glycolysis to sustain tumor growth at maximal levels (2, 37, 38).

**Figure 5:**
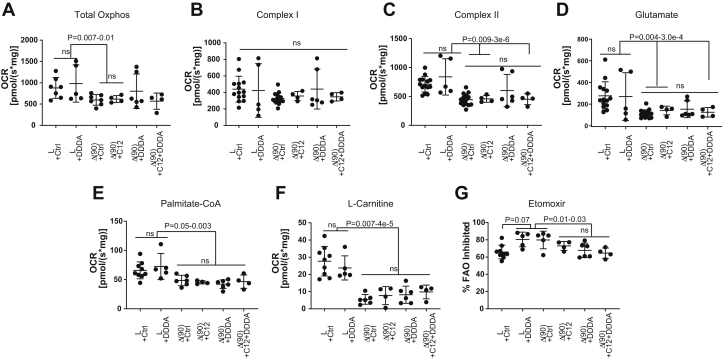
**Oxygen consumption rates (OCRs) in livers and tumors from mice maintained on standard, C12-, or DDDA-enriched diets.** Mitochondria were isolated from livers and hepatoblastomas of mice maintained on the indicated diets (Fig. 2*A*), and OCRs were determined as previously described (18, 19, 20, 21, 22). *A,* total oxidative phosphorylation (Oxphos) in response to the addition of cytochrome c, malate, ADP, pyruvate, glutamate, and succinate. *B* and *C,* complex I and complex II activities activity. *D,* the glutamate response was determined after the addition of cytochrome c, malate, ADP, and pyruvate. *E,* OCRs in response to palmitoyl-CoA were determined following the addition of cytochrome c, malate, ADP, and l-carnitine. *F,* OCRs in response to l-carnitine were determined following the addition of cytochrome c, malate, and ADP. *G,* the fraction of β-FAO that was Cpt1a dependent was determined following the addition of etomoxir to palmitoyl-CoA– and l-carnitine–supplemented reactions as described in *E.* DDDA, dodecanedioic acid.

To determine whether DDDA diets might be contributing to HB inhibition *via* a similar mechanism, we compared oxygen consumption rates (OCRs) of partially purified mitochondria from the livers and tumors of the cohorts shown in Figure 1*A* in response to several standard and anaplerotic TCA cycle substrates (Fig. 5, *A*–*C*) (18, 19, 22, 38). Consistent with our previous findings, total OCRs were significantly reduced in control diet tumors relative to control diet livers (Fig. 5*A*) with a somewhat greater contribution being made by complex II in each case (Fig. 5, *B* and *C*). This was consistent with the marked reduction in mitochondrial mass documented in murine HBs and HCCs (18, 19, 22, 38). Interestingly, each of two “outlier points” with the highest OCRs originated from mice with the longest survival. A determination of mitochondrial mass in these two tumors, based on quantification of their mitochondrial DNA content, did not show any significant differences relative to other members of their cohort indicating that the normalization of their responses was due to a more efficient utilization of substrates.

Rather than increasing glutaminolysis as occurs in many tumors and cancer cell lines (1, 5), HBs actually decrease this activity indicating that they are not reliant on the anaplerotic delivery of glutamate-derived α-ketoglutarate (19, 22, 38). This remained the case in HBs from mice on C12, DDDA, and C12 + DDDA diets (Fig. 5*D*).

Finally, we examined β-FAO in two ways. First, we measured the response to palmitoyl-CoA following the addition of all necessary substrates including l-carnitine (Fig. 5*E*). This allowed us to ascertain the inherent activity of mitochondrial β-FAO in a nonsubstrate-limiting manner. Second, we measured the response to l-carnitine in the absence of exogenous palmitoyl-CoA as an indirect way of quantifying the pre-existing stores of fatty acyl-CoA derivatives that were available for immediate Cpt1a-mediated transport into the mitochondrial matrix (Fig. 5*F*). All tumors, regardless of the diets upon which the animals had been maintained, showed similar responses. Finally, the loss of palmitoyl-CoA oxidation in response to etomoxir-mediated Cpt1a inhibition was less pronounced in tumors from C12 and/or DDDA diet–maintained mice (Fig. 5*G*). This suggested that these HBs were more reliant on short chain fatty acids whose mitochondrial oxidation is Cpt1a independent. Alternatively, they may have adapted to the high levels of succinate provided by C12/DDDA catabolism (Fig. 2*A*). Subtle alterations in complex I function, as suggested by the dysregulation of several transcripts encoding complex I subunits (Fig. 4*E*) in DDDA diet–maintained mice, might also have contributed. In sum, these results indicated that, despite the large-scale oxidation of C12 and DDDA by the ω-/peroxisomal-FAO pathway and its provision of TCA substrates such as succinate (Fig. 2*A*), overall mitochondrial function in response to standard substrates was, with minor exceptions, largely unaffected.

## Discussion

The optimization of tumor growth and survival in response to metabolic reprogramming is well documented and theoretically affords unique therapeutic opportunities, including those based on dietary manipulation (1, 2, 4, 6, 7, 40, 41, 42). The latter include caloric restriction or glucose deprivation, which reduce Warburg-type respiration and temper insulin and/or insulin-like growth factor signaling (4, 43, 44, 45, 46). The ensuing changes in the microenvironmental nutrient supply can impact tumor cell signaling pathways, growth, and chemotherapeutic sensitivity (9, 47, 48, 49).

We previously exploited a mouse model of HCC in which preservation of the Randle cycle allowed the suppression of glycolysis by β-FAO in response to HFDs, thereby impairing tumor growth and extending survival (38). A confirmatory analysis of primary human cancers showed that at least six tumor types with high β-FAO:glycolysis transcript ratios were also associated with significantly longer survival (38). Nondietary approaches to achieving similar results include the direct pharmacologic inhibition of glycolysis, glutaminolysis, and fatty acid synthesis or the specific targeting of mutant enzymes that generate oncometabolites (14, 15, 50, 51).

The common feature of the aforementioned approaches is that they restrict the overproduction of metabolites that are needed by transformed cells to maximize proliferation and/or block differentiation. For example, in addition to generating ATP, Warburg respiration produces glucose-6-phosphate, the initial substrate for the pentose phosphate pathway, as well as reducing equivalents in the form of NAD(P)H. It further provides 3-phosphoglycerate for serine, glycine, and purine nucleotide biosynthesis and pyruvate, which furnishes alanine and other amino acids and the anaplerotic TCA cycle substrate oxaloacetate. Similarly, glutaminolysis is an anaplerotic source of α-ketoglutarate, another TCA cycle intermediate, which supplies aspartate and asparagine as well as citrate *via* reductive carboxylation (2, 47, 48, 49, 50, 51, 52). Finally, the neomorphic activity of isocitrate dehydrogenase missense mutants generates the novel oncometabolite 2-hydroxyglutarate, which maintains the undifferentiated state of the blast cell population in acute myelogenous leukemia (53, 54). Inhibition of this mutant enzyme can induce terminal differentiation and hematologic remission (54, 55).

A limitation of the aforementioned approaches is that they typically reduce tumor cell proliferation without necessarily eliminating the malignant clone. Compensatory metabolic adaptation can also circumvent such therapies by reducing the initial dependence on the targeted pathway (55, 56). Cancer stem cells may also be less susceptible to such interventions either because of their inherent quiescence and/or because of their different metabolic dependencies (16). Finally, pharmacologic inhibition of cancer cell metabolic pathways is often limited by a narrow therapeutic window since the targets are seldom unique and are invariably active in normal cells as well.

The approach taken in the current work differs from those described previously in that it capitalizes on the tumor-specific loss of expression of a specific enzyme. This approach was inspired by the metabolic disorder type I hereditary tyrosinemia, in which the absence of fumarylacetoacetate hydrolase leads to the accumulation of the hepatotoxic metabolite fumarylacetoacetate and ultimately to liver failure (57). The acquired Ehhadh deficiency of HBs is conceptually similar except for its confinement to the transformed hepatocyte population. The fact that both normal and tumor-bearing mice tolerate high dietary burdens of C12 and DDDA, either alone or in combination and without evidence of overt toxicity (25), speaks to a high therapeutic index that is difficult to achieve with other approaches.

Tumor inhibition by either C12 and DDDA (Fig. 1) supports the idea that this is due to the accumulation of ω-/peroxisomal-FAO pathway–related metabolites–most likely DDDA itself–rather than to off-target effects. The fact that C12 and DDDA were not additive in extending survival also supports this idea and further indicates that the maximal antitumor effect is achieved with either compound. This was somewhat surprising in the case of C12 given the marked downregulation of the two cytochrome P450 enzymes (Cyp4a10 and Cyp4a14) that participate in the proximal catabolism of C12 (Fig. 2, *A*–*C*). Sufficiently toxic levels of DDDA must therefore still accumulate in the face of C12-enriched diets despite the dramatic suppression of Cyp4a10 and Cyp4a14. The ensuing hepatotoxicity thus likely only requires levels of DDDA that can be generated by the reduced levels of these two cytochromes. The inability of C12 and DDDA to completely eliminate all tumor cells likely reflects their residual Ehhadh levels and/or their eventual adaptation to otherwise toxic levels of DDDA (Fig. 2, *C* and *G*).

The downregulation of Ehhadh and other ω-/peroxisomal-FAO pathway–related transcripts seen in murine HBs (Fig. 2*B*) was recapitulated in Myc-driven murine HCCs, human HBs, and subsets of several other human tumor types (Fig. 2, *D*–*F*) and closely correlated with protein levels (Fig. 2, *C* and *G*). In some cases, Ehhadh transcript downregulation was particularly dramatic with tumors showing >95% suppression relative to corresponding normal tissue (Fig. 2*E*). It is currently not clear whether loss of Ehhadh in other tumors would necessarily render them susceptible to C12 or DDDA or whether their toxic effects are hepatocyte specific. In mice with germ-line *ehhadh* gene knockout, the most striking effect of dietary supplementation is massive hepatic necrosis with no toxicity having been reported in other tissues (25). However, it is certainly conceivable that the rapidity with which lethal hepatic failure develops could mask more protracted consequences of DDDA accumulation elsewhere. Testing these compounds directly in nonhepatic cancers with documented downregulation of the ω-/peroxisomal-FAO pathway will be needed to establish the broader efficacy of this dietary approach.

The direct relationship between mitochondrial β-FAO– and ω-/peroxisomal-FAO–related transcript downregulation observed in murine and human HBs (Fig. 2, *B* and *D*) was seen in other cancer types, although, not unexpectedly, the correlations were somewhat more modest when tumors were considered as single homogeneous groups (Fig. 3, *A*–*F*). However, in six cases, tumor subsets with the lowest levels of these transcripts were associated with shorter survivals relative to those with the highest levels. In three cancer types, an inverse relationship was seen between the transcripts and those encoding enzymes of the glycolytic pathway (Fig. 3*G*) but in only two of these were higher levels of glycolytic transcript expression associated with shorter survival. As already discussed with regard to HB, this inverse correlation between FAO and glycolysis is consistent with the preservation of Randle cycle–type regulation and agrees with previous reports demonstrating that survival for some cancers is inversely correlated with glycolytic pathway activity (58, 59, 60, 61, 62, 63, 64). The prolongation of survival by normalizing β-FAO with HFDs and by reciprocally suppressing Warburg-type respiration (38) suggests that this approach could potentially be combined with C12- or DDDA-enriched diets to capitalize on the additional ω-/peroxisomal-FAO pathway vulnerability described here.

The eventual progression of tumors in all DDDA-treated mice, despite impressive initial responses (Fig. 2, *B*–*D*), was consistent with numerous other studies involving dietary interventions administered either singly or in combination with chemotherapeutic agents (4, 38, 43, 45, 65, 66). Resistant HBs that failed to re-express Ehhadh and Cyp4a10 (Fig. 4*B*) altered the expression of only 129 genes, nearly 90% of which were downregulated relative to those of DDDA-sensitive tumors (Fig. 4*E* and Tables S1 and S2). Despite its small size, this transcript collection may be an overestimate since we used three independent genomic analysis methods to identify its members and did not require that they necessarily agree. In some cases, we also liberalized the false discovery rate (FDR) and instead relied on *p* values of <0.001. Given these caveats, perhaps the most surprising finding of this analysis was the nature of the gene expression differences between the two tumor groups. Only 10 transcripts encoded metabolic enzymes or those functioning to counter oxidative/electrophilic stress or redox imbalances. This suggested that the development of this form of DDDA resistance might involve post-transcriptional changes in pathways unrelated to ω-/peroxisomal-FAO that efficiently reduce intracellular DDDA levels. This might involve changes in the ECM as evidenced by the altered expression of 28 transcripts encoding several of its components, including enzymes such as matrix metalloproteases or several cell–cell or cell–matrix adhesion molecules. Such changes often affect chemotherapeutic sensitivity and could impact the efficiency with which DDDA traverses the vasculature and enters tumor cells (67, 68, 69, 70, 71, 72). The downregulation of transcripts encoding endothelial cell–related proteins, such as Eng, Angptl7, Esam, and Scarf2, might further contribute to alterations in blood vessel permeability.

More than half the transcripts listed in Tables S1 and S2 correlated with long-term and favorable survival among a group of nearly 8000 samples representing 17 general cancer types from The Cancer Genome Atlas (Table S3). These associations were concordant in nearly 80% of the cases and were particularly prominent in renal cancers and HCCs. Combined with the selective differential expression of transcripts functioning in transcriptional regulation, metabolism, and extracellular environment, these findings strongly suggest that resistant tumors represent a more aggressive form of HB that arises as a consequence of the reprogramming of a limited and functionally diverse set of pathways.

In most cases, C12- and/or DDDA-supplemented diets did not affect the OCRs of livers or tumors in response to different substrates (Fig. 5). This is perhaps to be expected given that peroxisomal β-FAO does not generate energy directly although it does provide select TCA cycle substrates such as succinate and AcCoA (Fig. 2*A*) (31). Two notable outliers to this, however, were seen in response to the full complement of nonfatty acid–related TCA cycle substrates (Fig. 5, *A*–*C*), and both these were associated with mice having the longest survival. Mitochondria from these two tumors showed OCR responses at the upper end of the range seen in control livers and were reminiscent of those seen in livers or in tumors from animals maintained on HFDs (38). However, the mitochondrial DNA content of these tumors was not increased relative to that of the other tumors. The basis for this apparent normalization and the degree to which it affects survival is currently unknown although it provides additional reason to believe that a modification of our previously used HFD regimen so as include C12 might provide additional survival benefit by targeting both mitochondria and peroxisomes (38).

Collectively, our results show that the severe acquired Ehhadh deficiency of HBs, HCCs, and certain other cancers represents a distinct, tumor-specific, and actionable metabolic susceptibility. The selective reduction of this enzyme causes the aberrant accumulation of DDDA, a highly toxic product of ω-/peroxisomal-FAO (25), and extensive tumor cell killing. On the other hand, normal liver parenchyma and other tissues are spared from any discernible toxicity as they maintain levels of Ehhadh sufficient to metabolize DDDA (Fig. 2*A*). The ability to achieve significant antitumor responses with this dietary approach potentially offers simple, inexpensive, well-tolerated, and non–cross-resistant therapeutic options that would be compatible with more traditional treatments and for individuals who were not candidates for them.

## Experimental procedures

### HB generation and diets

Hydrodynamic tail vein injections of Sleeping Beauty vectors encoding β-catenin and YAP^S127A^ were performed as previously described in 6- to 7-week old FVB mice (Jackson Labs) (19, 20, 21, 73). Mice were initially monitored weekly and at least thrice weekly after tumors first became detectable. Animals were sacrificed when tumors reached a size of 2 cm in any dimension or when signs of stress were observed. All injections, monitoring procedures, and other routine care and husbandry were approved by The University of Pittsburgh Department of Laboratory and Animal Resources and the Institutional Animal Care and Use Committee. Diets consisted of standard animal chow which, where indicated, were supplemented with 10% (w/w) C12 in the form of trilauryl glycerate and/or 10% DDDA (Sigma–Aldrich, Inc). Tumor size and appearance were monitored by The UPMC Children's Hospital Core Animal Imaging Facility using a 7-Teslar micro-MRI system (BioSpin 70/30; Bruker).

### Immunoblotting and immunohistochemistry

Tissue lysates were prepared for immunoblotting as previously described (18, 19, 74). Antibodies used included those against Ehhadh (1:500 dilution, #ab93173; Abcam, Inc), Cyp4a10 (1:1000 dilution, #PA3-033; Invitrogen/Thermo-Fisher, Inc), serpin E1/PAI-1 (1:1200 dilution, #AF3828; R&D Systems, Inc/Fisher Scientific), and GAPDH (1:10,000 dilution, G8795; Sigma–Aldrich).

Immunohistochemistry for Ehhadh was performed on tissue microarrays of 40 HBs that were maintained as paraffin-embedded samples at UPMC Children's Hospital of Pittsburgh. All tissues were deidentified and were obtained with the approval of the University of Pittsburgh's Institutional Review Board guidelines and were in accordance with the Declaration of Helsinki principles. Immunostaining was performed in the core histology laboratory of The University of Pittsburgh Liver Research Center using standard methodologies with the aforementioned anti-Ehhadh antibody (1:200 dilution) followed by secondary immunoperoxidase staining. Poststaining evaluation was undertaken by a board-certified pathologist and member of The Children's Oncology Liver Tumor Committee (S. R.).

### Respirometry/metabolic studies

OCRs were performed on 50 to 100 mg of disrupted tissue immediately after sacrifice as previously described (18, 19, 20, 21, 22, 38, 74). Studies were performed with an Oroboros Oxygraph 2k instrument (Oroboros Instruments, Inc) (18, 19, 73). Baseline OCRs were determined in 2 ml samples in Mir05 buffer following the addition of cytochrome c (final concentration = 10 μM), malate (2 mM), ADP (5 mM), pyruvate (5 mM), and glutamate (10 mM). Together, these substrates provided an estimate of the activity of complex I. Succinate was then added (10 mM final concentration) to determine the additional contribution provided by complex II. Complex I inhibitor rotenone was then added (0.5 μM final concentration) to calculate the proportional contributions of complexes I and II. Activities were normalized to total protein.

### Quantification of transcripts encoding ω-/peroxisomal-FAO in human cancers

RNA-Seq studies were performed as previously described (18, 20, 21). Briefly, total RNA was extracted from five replicate samples of each indicated group of tissues using Qiagen RNAeasy columns followed by DNase digestion as directed by the supplier (Qiagen, Inc). RNA samples were assessed for purity and integrity with an Agilent 2100 Bioanalyzer (Agilent Technologies), and only samples with RNA integrity number values >8.5 were processed further. Sequencing reactions were prepared using a NEBNext Ultra Directional RNA Library Prep kit according to the recommendations of the vendor (New England Biolabs). Sequencing was performed on a NovaSeq 600 instrument (Illumina, Inc) by Novagene, Inc. Gene expression was calculated as previously described (18, 20, 22) as fragments per kilobase million, normalized across samples, and statistically compared between experimental groups with an FDR-adjusted q value of <0.05. Raw and processed original data have been deposited in the National Center for Biotechnology Information Gene Expression Omnibus database. Ingenuity Pathway Analysis (Qiagen) software was used to categorize expressed genes into different pathways and was adjusted for FDR using a Bonferroni–Hochberg correction.

In the case of human transcripts, expression values (fragments per kilobase of transcript per million mapped reads upper quartile) for all relevant transcripts were downloaded from the Genomic Data Commons-PANCAN dataset via the University of California Santa Cruz Xenabrowser (xena.ucsc.edu). In cases where a patient had more than one sample of the same type, the expression values were averaged. Significance of expression differences between matched primary tumor and normal tissue samples was assessed for each cancer using Welch's *t* test where the members of the groups were the base-two logarithms of the expression values for each patient. The average tumor to liver expression ratio for each cancer was calculated by taking the ratio of the average expression across all tumor samples and dividing by the average for normal tissue samples.

## Data availability

Raw and processed RNA-Seq data were deposited in the National Center for Biotechnology Information Gene Expression and are accessible through Gene Expression Omnibus (https://www.ncbi.nlm.nim.gov/geo) at accession number GSE156545. All remaining data are contained within the article.

## Conflict of interest

The authors declare that they have no conflicts of interest with the contents of this article.
