# Supplementary material for: Acquired deficiency of peroxisomal dicarboxylic acid catabolism is a metabolic vulnerability in hepatoblastoma
Source: J Biol Chem. 2021 Jan 13;296:100283. doi: 10.1016/j.jbc.2021.100283 (PMC7948956; doi:10.1016/j.jbc.2021.100283)
Supplement: Figures and Tables [file mmc1.docx]

## Supporting Information

**Acquired Deficiency of Peroxisomal Dicarboxylic Acid Catabolism is a Metabolic Vulnerability in Hepatoblastoma**

Huabo Wang^1^, Jie Lu^1^, Xiaoguang Chen^1,2^, Marie Schwalbe^1^, Joanna E. Gorka^1^, Jordan A. Mandel^1^, Jinglin Wang^1,3^, Eric S. Goetzman^4^, Sarangarajan Ranganathan^5^, Steven Dobrowolski^4^, Edward V. Prochownik^1,6,7,8,^*

^1^Division of Hematology/Oncology, Department of Pediatrics UPMC Children’s Hospital of Pittsburgh, Pittsburgh, PA; ^2^School of Animal Science and Technology, Henan University of Science and Technology, People’s Republic of China; ^3^Central South University Xiangya School of Medicine, [Changsha](https://en.wikipedia.org/wiki/Changsha), [Hunan](https://en.wikipedia.org/wiki/Hunan), People’s Republic of China; ^4^Division of Medical Genetics, Department of Pediatrics, UPMC Children’s Hospital of Pittsburgh, Pittsburgh, PA; ^5^Department of Pathology, Cincinnati Children’s Hospital, Cincinnati, OH; ^6^The Hillman Cancer Center, The University of Pittsburgh Medical Center; ^7^The Pittsburgh Liver Research Institute; ^8^The Department of Microbiology and Molecular Genetics, The University of Pittsburgh Medical Center, Pittsburgh, PA

*Corresponding author: Edward V. Prochownik, MD, PhD

E-mail: [procev@chp.edu](mailto:procev@chp.edu)

**Running title:** Ehhadh deficiency is a metabolic vulnerability

**Keywords:** cancer metabolism; Ehhadh, fatty acid oxidation; hepatocellular carcinoma; metabolic re-programming; oxidative phosphorylation; Warburg effect, peroxisome

**Supplementary Figures**


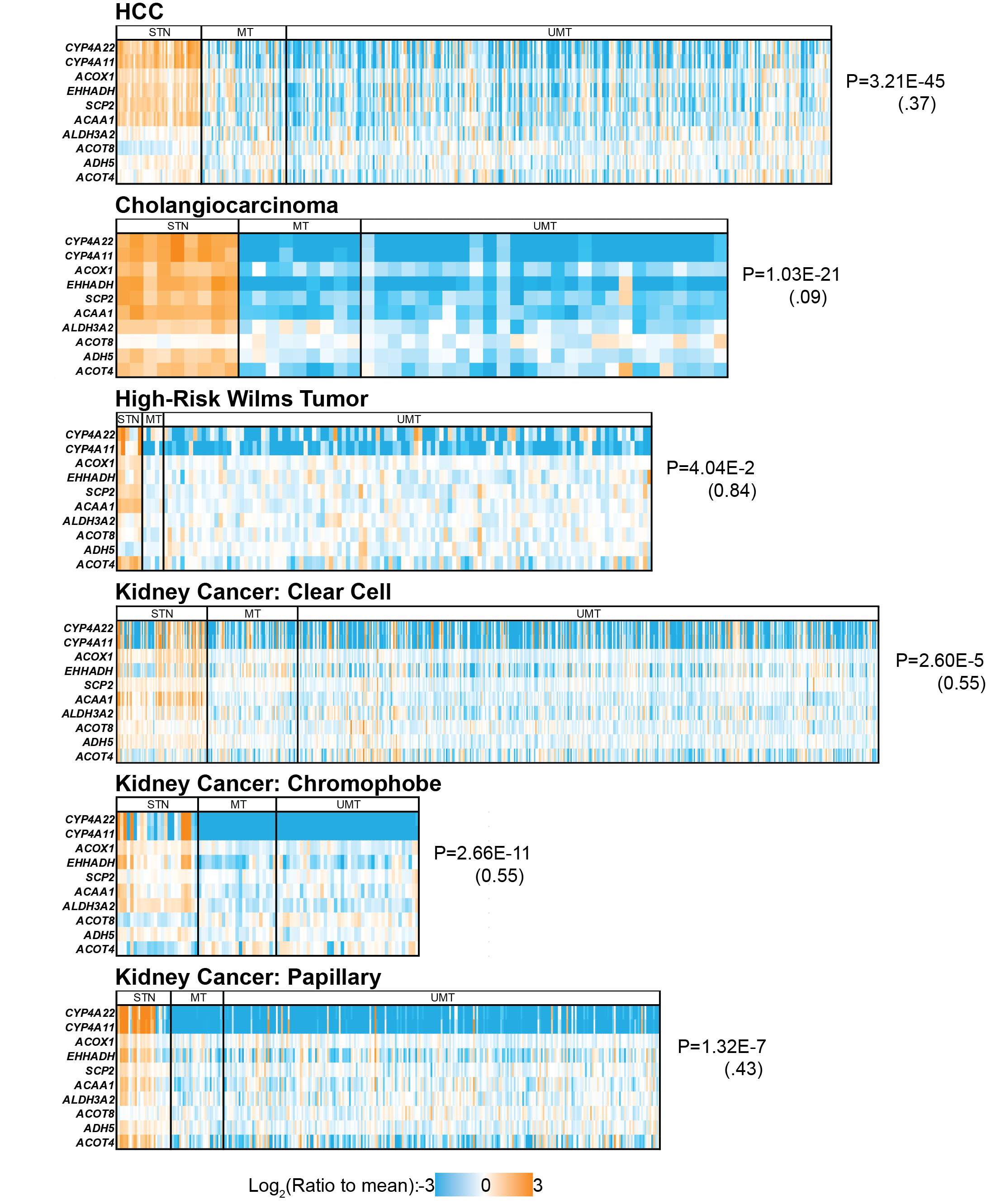


**Supplementary Figure 1. Down regulation of the ω-/peroxisomal-FAO pathways in human cancers.**  Expression of ω-/peroxisomal FAO pathway transcripts are shown in primary tumors from the indicated cancers. Matched primary tissues are shown at the extreme left of each heat map. Numbers to the right in parentheses indicate the fractional expression of the transcripts as a group in all tumors and P values relative to the expression of the corresponding transcripts in matched control tissues. STN = Solid Tissue Normal; MT=Matched Tumor; UMT= Unmatched Tumor


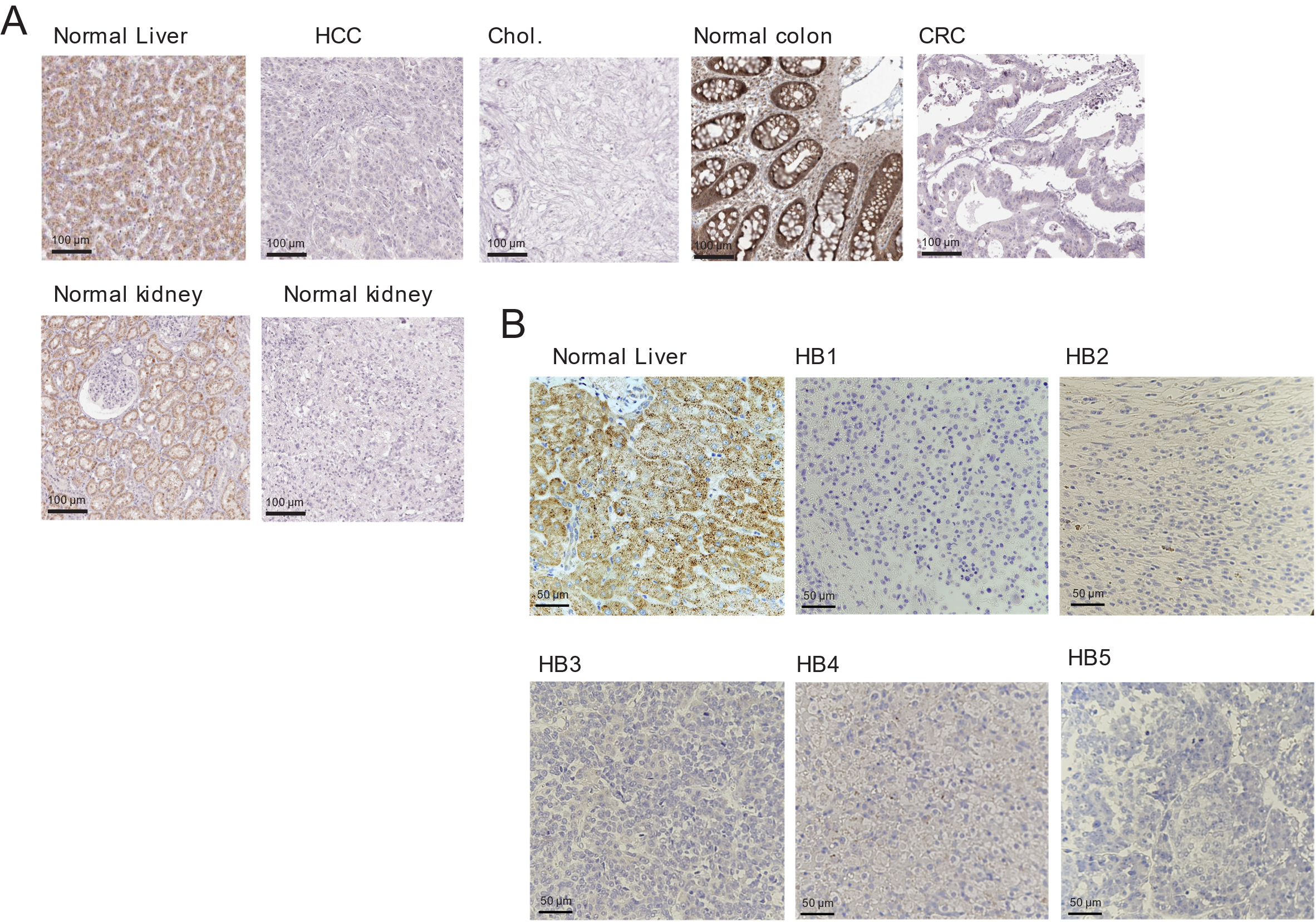


**Supplementary Figure 2. Select adult cancers and HBs show reduced Ehhadh protein expression.** *A.* Typical examples of Ehhadh expression detected by IHC in normal adult liver, colon and kidney and tumors arising from these organs were obtained from The Human Protein Atlas (<https://www.proteinatlas.org/ENSG00000113790-EHHADH/pathology/>) HCC=hepatocellular carcinoma, Chol=cholangiocarcinoma, CRC=colo-rectal cancer. *B.* Ehhadh IHC obtained from a tissue microarray containing 40 HBs, 25 of which stained negative for Ehhadh, compared to normal control liver tissue or tumor-adjacent non-transformed liver.

**Supplementary Tables**

**Supplementary Table 1. Gene expression differences in HBs arising in mice maintained on DDDA diets for 3 wks (sensitive group) versus >20 wks (resistant group).** q<0.05 in all cases. Negative values indicate down-regulation in the latter group relative to the former group. In the first column, transcripts encoding proteins involved in extracellular matrix architecture and its re-modeling or cell adhesion are highlighted in yellow, those encoding transcription factors or co-activators are highlighted in red and those encoding enzymes that regulate metabolism or maintain redox homeostasis are highlighted in aqua. In the second column, transcripts encoding known or putative tumor suppressors are highlighted in blue and those encoding known or putative oncoproteins are highlighted in green. Note that dual functionality has been attributed to two transcripts (Add3 and Lum).

| Name | Identifier | Fold difference (resistant vs. sensitive) | P value | Resistant mean | Sensitive mean |  |  |  |
| --- | --- | --- | --- | --- | --- | --- | --- | --- |
| Klhl13 | [67455](https://www.ncbi.nlm.nih.gov/entrez/query.fcgi?db=gene&cmd=Retrieve&dopt=full_report&list_uids=67455) | 4.36 | 1.35E-05 | 191.20 | 43.89 |  |  | Transcription factor |
| Hmgb1 | 15289 | -1.37 | 2.00E-02 | 87.31 | 119.67 |  |  | Metabolism |
| Hbb-bt | [101488143](https://www.ncbi.nlm.nih.gov/entrez/query.fcgi?db=gene&cmd=Retrieve&dopt=full_report&list_uids=101488143) | -6.54 | 1.88E-06 | 31.80 | 208.07 |  |  | Extracellular matrix remodeling |
| Gpx3 | [14778](https://www.ncbi.nlm.nih.gov/entrez/query.fcgi?db=gene&cmd=Retrieve&dopt=full_report&list_uids=14778) | -4.76 | 2.97E-05 | 28.28 | 134.52 |  |  | Known/putative tumor suppressor |
| Diablo | 66593 | -1.34 | 3.00E-02 | 22.03 | 29.46 |  |  | Known/putative oncogene |
| Emilin1 | [100952](https://www.ncbi.nlm.nih.gov/entrez/query.fcgi?db=gene&cmd=Retrieve&dopt=full_report&list_uids=100952) | -3.98 | 1.13E-04 | 19.79 | 78.77 |  |  |  |
| Mgp | [17313](https://www.ncbi.nlm.nih.gov/entrez/query.fcgi?db=gene&cmd=Retrieve&dopt=full_report&list_uids=17313) | -7.46 | 2.66E-06 | 11.90 | 88.84 |  |  |  |
| Afp | [11576](https://www.ncbi.nlm.nih.gov/entrez/query.fcgi?db=gene&cmd=Retrieve&dopt=full_report&list_uids=11576) | -9.88 | 2.68E-05 | 11.30 | 111.72 |  |  |  |
| Lbh | [77889](https://www.ncbi.nlm.nih.gov/entrez/query.fcgi?db=gene&cmd=Retrieve&dopt=full_report&list_uids=77889) | -4.03 | 5.74E-05 | 9.82 | 39.54 |  |  |  |
| Add3 | [27360](https://www.ncbi.nlm.nih.gov/entrez/query.fcgi?db=gene&cmd=Retrieve&dopt=full_report&list_uids=27360) | -2.68 | 5.62E-05 | 9.23 | 24.78 |  |  |  |
| Esam | [69524](https://www.ncbi.nlm.nih.gov/entrez/query.fcgi?db=gene&cmd=Retrieve&dopt=full_report&list_uids=69524) | -2.99 | 6.99E-05 | 7.31 | 21.88 |  |  |  |
| Lum | [17022](https://www.ncbi.nlm.nih.gov/entrez/query.fcgi?db=gene&cmd=Retrieve&dopt=full_report&list_uids=17022) | -5.59 | 7.67E-06 | 6.89 | 38.56 |  |  |  |
| Ctla2a | [13024](https://www.ncbi.nlm.nih.gov/entrez/query.fcgi?db=gene&cmd=Retrieve&dopt=full_report&list_uids=13024) | -5.70 | 4.23E-05 | 6.66 | 38.01 |  |  |  |
| Tm4sf1 | [17112](https://www.ncbi.nlm.nih.gov/entrez/query.fcgi?db=gene&cmd=Retrieve&dopt=full_report&list_uids=17112) | -3.45 | 4.73E-06 | 4.98 | 17.22 |  |  |  |
| Plxdc2 | [67448](https://www.ncbi.nlm.nih.gov/entrez/query.fcgi?db=gene&cmd=Retrieve&dopt=full_report&list_uids=67448) | -5.40 | 2.01E-05 | 4.58 | 24.78 |  |  |  |
| Tagln | [21345](https://www.ncbi.nlm.nih.gov/entrez/query.fcgi?db=gene&cmd=Retrieve&dopt=full_report&list_uids=21345) | -7.24 | 4.26E-06 | 4.55 | 32.95 |  |  |  |
| Bag2 | [213539](https://www.ncbi.nlm.nih.gov/entrez/query.fcgi?db=gene&cmd=Retrieve&dopt=full_report&list_uids=213539) | -2.68 | 6.80E-05 | 4.02 | 10.81 |  |  |  |
| Mmp2 | [17390](https://www.ncbi.nlm.nih.gov/entrez/query.fcgi?db=gene&cmd=Retrieve&dopt=full_report&list_uids=17390) | -7.60 | 4.43E-06 | 3.33 | 25.35 |  |  |  |
| Mxra8 | [74761](https://www.ncbi.nlm.nih.gov/entrez/query.fcgi?db=gene&cmd=Retrieve&dopt=full_report&list_uids=74761) | -4.28 | 9.39E-05 | 3.29 | 14.10 |  |  |  |
| Gm8203 | [666634](https://www.ncbi.nlm.nih.gov/entrez/query.fcgi?db=gene&cmd=Retrieve&dopt=full_report&list_uids=666634) | -11.33 | 1.72E-13 | 3.01 | 34.10 |  |  |  |
| Eln | [13717](https://www.ncbi.nlm.nih.gov/entrez/query.fcgi?db=gene&cmd=Retrieve&dopt=full_report&list_uids=13717) | -5.23 | 3.87E-06 | 2.84 | 14.90 |  |  |  |
| Prrx1 | [18933](https://www.ncbi.nlm.nih.gov/entrez/query.fcgi?db=gene&cmd=Retrieve&dopt=full_report&list_uids=18933) | -9.98 | 6.51E-12 | 1.95 | 19.54 |  |  |  |
| Robo1 | [19876](https://www.ncbi.nlm.nih.gov/entrez/query.fcgi?db=gene&cmd=Retrieve&dopt=full_report&list_uids=19876) | -8.11 | 3.66E-10 | 1.40 | 11.40 |  |  |  |
| S100a9 | [20202](https://www.ncbi.nlm.nih.gov/entrez/query.fcgi?db=gene&cmd=Retrieve&dopt=full_report&list_uids=20202) | -18.31 | 4.92E-06 | 1.31 | 24.12 |  |  |  |
| Slit3 | [20564](https://www.ncbi.nlm.nih.gov/entrez/query.fcgi?db=gene&cmd=Retrieve&dopt=full_report&list_uids=20564) | -7.77 | 1.19E-04 | 1.11 | 8.67 |  |  |  |
| Ugt2b37 | [112417](https://www.ncbi.nlm.nih.gov/entrez/query.fcgi?db=gene&cmd=Retrieve&dopt=full_report&list_uids=112417) | -7.31 | 1.05E-04 | 1.02 | 7.50 |  |  |  |
| Stk39 | [53416](https://www.ncbi.nlm.nih.gov/entrez/query.fcgi?db=gene&cmd=Retrieve&dopt=full_report&list_uids=53416) | -4.16 | 7.63E-05 | 0.98 | 4.11 |  |  |  |
| Degs2 | [70059](https://www.ncbi.nlm.nih.gov/entrez/query.fcgi?db=gene&cmd=Retrieve&dopt=full_report&list_uids=70059) | -7.58 | 5.37E-06 | 0.84 | 6.43 |  |  |  |
| Cbr2 | [12409](https://www.ncbi.nlm.nih.gov/entrez/query.fcgi?db=gene&cmd=Retrieve&dopt=full_report&list_uids=12409) | -5.07 | 4.65E-05 | 0.70 | 3.61 |  |  |  |
| S100a8 | [20201](https://www.ncbi.nlm.nih.gov/entrez/query.fcgi?db=gene&cmd=Retrieve&dopt=full_report&list_uids=20201) | -20.82 | 3.49E-06 | 0.70 | 14.77 |  |  |  |
| Mmp23 | [26561](https://www.ncbi.nlm.nih.gov/entrez/query.fcgi?db=gene&cmd=Retrieve&dopt=full_report&list_uids=26561) | -5.35 | 6.03E-05 | 0.61 | 3.32 |  |  |  |
| Snhg11 | [319317](https://www.ncbi.nlm.nih.gov/entrez/query.fcgi?db=gene&cmd=Retrieve&dopt=full_report&list_uids=319317) | -12.31 | 2.04E-06 | 0.60 | 7.47 |  |  |  |
| Setbp1 | 240427 | -4.03 | 9.50E-05 | 0.60 | 2.47 |  |  |  |
| Serpina12 | [68054](https://www.ncbi.nlm.nih.gov/entrez/query.fcgi?db=gene&cmd=Retrieve&dopt=full_report&list_uids=68054) | -10.75 | 1.27E-06 | 0.45 | 4.89 |  |  |  |
| Camp | [12796](https://www.ncbi.nlm.nih.gov/entrez/query.fcgi?db=gene&cmd=Retrieve&dopt=full_report&list_uids=12796) | -31.69 | 4.13E-05 | 0.28 | 9.10 |  |  |  |
| Hspb7 | [29818](https://www.ncbi.nlm.nih.gov/entrez/query.fcgi?db=gene&cmd=Retrieve&dopt=full_report&list_uids=29818) | -9.53 | 6.56E-05 | 0.26 | 2.56 |  |  |  |
| Angptl7 | [654812](https://www.ncbi.nlm.nih.gov/entrez/query.fcgi?db=gene&cmd=Retrieve&dopt=full_report&list_uids=654812) | -26.15 | 6.31E-07 | 0.21 | 5.63 |  |  |  |
| Baiap2l2 | [207495](https://www.ncbi.nlm.nih.gov/entrez/query.fcgi?db=gene&cmd=Retrieve&dopt=full_report&list_uids=207495) | -24.05 | 3.44E-07 | 0.18 | 4.39 |  |  |  |
| Psma8 | [73677](https://www.ncbi.nlm.nih.gov/entrez/query.fcgi?db=gene&cmd=Retrieve&dopt=full_report&list_uids=73677) | -11.88 | 1.90E-05 | 0.17 | 2.05 |  |  |  |
| Retnlg | [245195](https://www.ncbi.nlm.nih.gov/entrez/query.fcgi?db=gene&cmd=Retrieve&dopt=full_report&list_uids=245195) | -15.62 | 1.14E-04 | 0.14 | 2.30 |  |  |  |
| Bpifa1 | [18843](https://www.ncbi.nlm.nih.gov/entrez/query.fcgi?db=gene&cmd=Retrieve&dopt=full_report&list_uids=18843) | -564.28 | 2.57E-06 | 0.00 | 3.31 |  |  |  |

**Supplementary Table 2. Additional gene expression differences in HBs that were initially sensitive to 3 wks of DDDA-supplemented diets or that eventually became resistant after >20 wks.** In all cases, q>0.05, P<0.001. Transcripts encoding proteins involved in extracellular matrix architecture and maintenance or cell adhesion are highlighted in yellow, those encoding transcription factors or co-activators are highlighted in red and those encoding enzymes that regulate metabolism or maintenance redox homeostasis are highlighted in aqua. Highlighted color schemes are the same as those described in Supplementary Table 1.

| Name | Identifier | Fold difference (resistant vs. sensitive) | P value | Resistant mean | Sensitive mean |  |  |  |
| --- | --- | --- | --- | --- | --- | --- | --- | --- |
| Prex2 | 109294 | -2.05 | 8.59E-04 | 9.61 | 19.75 |  |  | Transcription factors |
| Col5a2 | 12832 | -4.56 | 6.43E-04 | 11.41 | 52.02 |  |  | Metabolism |
| Eng | 13805 | -2.42 | 8.70E-04 | 27.00 | 65.34 |  |  | Extracellular matrix remodeling |
| Tfpi | 21788 | -3.04 | 6.79E-04 | 2.28 | 6.96 |  |  | Known/putative tumor suppressor |
| Ambra1 | 228361 | 1.34 | 1.44E-02 | 55.43 | 41.44 |  |  | Known/putative oncogene |
| Srxn1 | 76650 | 2.23 | 7.86E-04 | 164.93 | 73.95 |  |  |  |
| Bpifa2 | 19194 | -324.42 | 1.75E-04 | 0.00 | 1.90 |  |  |  |
| Ncoa6 | 56406 | 1.50 | 2.40E-03 | 61.42 | 40.95 |  |  |  |
| Procr | 19124 | -3.71 | 7.40E-04 | 2.75 | 10.20 |  |  |  |
| Mmp9 | 17395 | -7.43 | 1.80E-04 | 0.92 | 6.87 |  |  |  |
| Ptgis | 19223 | -5.35 | 2.48E-04 | 1.10 | 5.93 |  |  |  |
| Arhgef26 | 622434 | 1.86 | 4.64E-04 | 58.98 | 31.78 |  |  |  |
| Rab25 | 53868 | -15.28 | 1.58E-04 | 0.08 | 1.32 |  |  |  |
| Ctsk | 13038 | -4.94 | 5.80E-04 | 1.07 | 5.31 |  |  |  |
| Olfml3 | 99543 | -3.83 | 1.56E-04 | 3.99 | 15.32 |  |  |  |
| Chil3 | 12655 | -11.32 | 2.34E-04 | 1.60 | 18.20 |  |  |  |
| Gdf6 | 242316 | -6.70 | 1.85E-04 | 0.88 | 5.95 |  |  |  |
| Rgs3 | 50780 | -2.67 | 7.18E-04 | 4.20 | 11.24 |  |  |  |
| Caap1 | 67770 | -1.50 | 4.99E-02 | 5.09 | 7.60 |  |  |  |
| Lepr | 16847 | -4.86 | 2.41E-04 | 1.93 | 9.41 |  |  |  |
| Fzd1 | 14362 | -2.50 | 1.54E-04 | 7.88 | 19.72 |  |  |  |
| Cenpa | 12615 | -2.24 | 9.87E-04 | 5.37 | 12.00 |  |  |  |
| Gpn1 | 74254 | -1.41 | 2.90E-02 | 9.72 | 13.68 |  |  |  |
| Mrpl33 | 66845 | -1.52 | 1.66E-03 | 16.12 | 24.52 |  |  |  |
| Fgfr3 | 14184 | -1.99 | 4.92E-04 | 19.02 | 37.85 |  |  |  |
| Med28 | 66999 | -1.46 | 3.95E-03 | 16.73 | 24.35 |  |  |  |
| Pdgfra | 18595 | -4.07 | 2.80E-04 | 4.32 | 17.60 |  |  |  |
| Smr3a | 20599 | -177.70 | 6.79E-04 | 0.00 | 1.03 |  |  |  |
| Enoph1 | 67870 | -1.47 | 4.81E-02 | 8.90 | 13.11 |  |  |  |
| Akr1b8 | 14187 | -3.66 | 7.94E-04 | 1.87 | 6.88 |  |  |  |
| Snca | 20617 | -4.30 | 4.53E-04 | 1.75 | 7.58 |  |  |  |
| Lhfpl4 | 269788 | 3.92 | 3.06E-04 | 13.16 | 3.36 |  |  |  |
| Mug2 | 17837 | 2.97 | 3.69E-04 | 40.94 | 13.76 |  |  |  |
| Fgf23 | 64654 | -10.89 | 4.33E-04 | 0.45 | 4.97 |  |  |  |
| Saa3 | 20210 | -7.66 | 7.12E-04 | 1.12 | 8.64 |  |  |  |
| Prss23 | 76453 | -4.78 | 2.06E-04 | 5.08 | 24.30 |  |  |  |
| Serpinh1 | 12406 | -3.17 | 8.98E-04 | 18.36 | 58.16 |  |  |  |
| Hbb-bs | 100503605 | -4.49 | 2.97E-04 | 375.73 | 1685.88 |  |  |  |
| Dkk3 | 50781 | -3.65 | 4.09E-04 | 3.51 | 12.84 |  |  |  |
| Gprc5b | 64297 | -3.82 | 3.79E-04 | 3.32 | 12.73 |  |  |  |
| Tgfb1i1 | 21804 | -4.22 | 6.61E-04 | 1.43 | 6.08 |  |  |  |
| Kcnq1 | 16535 | -4.95 | 3.12E-04 | 0.78 | 3.90 |  |  |  |
| Hand2 | 15111 | -3.70 | 9.00E-04 | 1.73 | 6.43 |  |  |  |
| Nkd1 | 93960 | -3.81 | 8.21E-04 | 6.56 | 25.01 |  |  |  |
| Fhod1 | 234686 | -1.99 | 4.31E-04 | 8.97 | 17.88 |  |  |  |
| Crispld2 | 78892 | -3.89 | 6.44E-04 | 1.07 | 4.16 |  |  |  |
| Loxl1 | 16949 | -4.73 | 3.12E-04 | 3.88 | 18.38 |  |  |  |
| Gsta1 | 14857 | 7.38 | 4.09E-04 | 21.18 | 2.87 |  |  |  |
| Gm3776 | 100042295 | 5.16 | 6.91E-04 | 10.88 | 2.11 |  |  |  |
| Ngp | 18054 | -20.99 | 2.63E-04 | 0.94 | 19.81 |  |  |  |
| Ltf | 17002 | -14.11 | 1.67E-04 | 0.88 | 12.54 |  |  |  |
| Tcf21 | 21412 | -5.44 | 3.14E-04 | 0.56 | 3.09 |  |  |  |
| Lama4 | 16775 | -3.80 | 7.45E-04 | 4.63 | 17.61 |  |  |  |
| Cdk1 | 12534 | -2.49 | 4.49E-04 | 11.65 | 28.97 |  |  |  |
| Col6a1 | 12833 | -4.06 | 4.73E-04 | 13.25 | 53.85 |  |  |  |
| Elane | 50701 | -14.44 | 8.95E-04 | 0.25 | 3.67 |  |  |  |
| Timp3 | 21859 | -3.45 | 7.15E-04 | 91.46 | 315.87 |  |  |  |
| Abcc3 | 76408 | 1.75 | 6.88E-03 | 370.45 | 211.22 |  |  |  |
| Dlx4 | 13394 | 4.19 | 4.20E-03 | 2.47 | 0.59 |  |  |  |
| Mrc2 | 17534 | -3.95 | 5.42E-04 | 4.90 | 19.37 |  |  |  |
| C1qtnf1 | 56745 | -2.42 | 3.15E-04 | 10.87 | 26.27 |  |  |  |
| Lamb1 | 16777 | -3.47 | 7.16E-04 | 8.27 | 28.70 |  |  |  |
| Gas1 | 14451 | -5.14 | 1.52E-04 | 1.46 | 7.51 |  |  |  |
| Fhit | 14198 | -2.81 | 9.14E-04 | 1.47 | 4.13 |  |  |  |
| Nid2 | 18074 | -2.31 | 5.79E-04 | 16.94 | 39.23 |  |  |  |
| Sncg | 20618 | -7.01 | 1.23E-04 | 0.81 | 5.73 |  |  |  |
| Mmrn2 | 105450 | -2.12 | 9.50E-04 | 8.65 | 18.33 |  |  |  |
| 1700011H14Rik | 67082 | -3.89 | 3.49E-04 | 2.77 | 10.83 |  |  |  |
| Klf12 | 16597 | 1.39 | 3.52E-02 | 52.90 | 38.19 |  |  |  |
| Mucl2 | 20770 | -244.13 | 4.18E-04 | 0.00 | 1.42 |  |  |  |
| Scarf2 | 224024 | -4.29 | 3.46E-04 | 2.84 | 12.22 |  |  |  |
| Hrg | 94175 | 2.88 | 1.31E-04 | 858.74 | 298.13 |  |  |  |
| Muc13 | 17063 | -4.52 | 5.43E-04 | 4.08 | 18.50 |  |  |  |
| Fstl1 | 14314 | -3.91 | 2.10E-04 | 9.18 | 35.95 |  |  |  |
| Zbtb20 | 56490 | 1.94 | 2.98E-03 | 250.41 | 128.80 |  |  |  |
| Tmem204 | 407831 | -2.86 | 6.46E-04 | 3.66 | 10.47 |  |  |  |
| Rab11fip3 | 215445 | 1.41 | 5.99E-03 | 50.31 | 35.73 |  |  |  |
| Fam234a | 106581 | 1.31 | 3.42E-02 | 104.22 | 79.48 |  |  |  |
| Tap1 | 21354 | 1.60 | 2.81E-02 | 40.47 | 25.33 |  |  |  |
| Ppic | 19038 | -3.71 | 2.01E-04 | 4.14 | 15.40 |  |  |  |
| Me2 | 107029 | -3.99 | 3.25E-04 | 5.55 | 22.12 |  |  |  |
| Scgb1a1 | 22287 | -50.54 | 4.14E-05 | 0.01 | 0.75 |  |  |  |
| Anxa1 | 16952 | -3.61 | 8.17E-04 | 7.31 | 26.44 |  |  |  |
| Prkg1 | 19091 | -3.92 | 1.60E-04 | 2.36 | 9.27 |  |  |  |
| Acta2 | 11475 | -5.55 | 2.21E-04 | 4.82 | 26.77 |  |  |  |
| Apln | 30878 | -8.00 | 2.60E-04 | 2.97 | 23.86 |  |  |  |
| Obp1a | 18249 | -279.36 | 3.03E-04 | 0.00 | 1.63 |  |  |  |
| Gm5938 | 546335 | -176.65 | 8.15E-04 | 0.00 | 1.03 |  |  |  |

**Supplementary Table 3. Correlation of gene expression differences from DDDA-resistant tumors with survival in human cancers.** Shown here are the genes from Supplementary Tables 1 and 2 whose expression correlated with survival in the cancer types listed in The Human Protein Atlas (<https://www.proteinatlas.org/humanproteome/pathology>). Tumors indicated in red are those in which low transcript expression correlated with significant shorter survival; those in green are those in which low transcript levels correlated with longer survival.

| Total Genes: 82 | |  |  |  |  |
| --- | --- | --- | --- | --- | --- |
| Total association with survival cancer type: 181 | | | | |  |
| Prognostic marker as **unfavorable(Red): 145 80.1% of total** | | | | | |
| Prognostic marker as **favorable(Green): 36 19.9% of total** | | | | | |
| Mouse gene name | Mouse gene ID | Human gene name | Human gene ID | Fold change: resistant vs. sensitive | Association with survival (cancer type) |
| Abcc3 | 76408 | ABCC3 | 8714 | 1.75 | **KIRC,KIRP,PAAD** |
| Acta2 | 11475 | ACTA2 | 59 | -5.55 | **KIRP** |
| Akr1b8 | 14187 | AKR1B10 | 57016 | -3.66 | **KIRC,KIRP,KICH,COAD** |
| Ambra1 | 228361 | AMBRA1 | 55626 | 1.34 | **KICH** |
| Anxa1 | 16952 | ANXA1 | 301 | -3.61 | **BLCA,THCA,UCEC** |
| Apln | 30878 | APLN | 8862 | -8.00 | **KIRC,KIRP,KICH,CESC,LUSC,LIHC** |
| Arhgef26 | 622434 | ARHGEF26 | 26084 | 1.86 | **LIHC** |
| Bag2 | 213539 | BAG2 | 9532 | -2.68 | **LIHC,THCA** |
| C1qtnf1 | 56745 | C1QTNF1 | 114897 | -2.42 | **KIRC,KICH,CESC** |
| Caap1 | 67770 | CAAP1 | 79886 | -1.50 | **KIRC,KIRP,OVCA** |
| Camp | 12796 | CAMP | 820 | -31.69 | **CESC** |
| Cdk1 | 12534 | CDK1 | 983 | -2.49 | **KIRC,KIRP,KICH,LIHC,LUSC,PAAD,CESC** |
| Cenpa | 12615 | CENPA | 1058 | -2.24 | **LIHC,LUSC,PAAD,UCEC** |
| Col5a2 | 12832 | COL5A2 | 1290 | -4.56 | **KICH,LUSC,BLCA** |
| Col6a1 | 12833 | COL6A1 | 1291 | -4.06 | **KIRC,KIRP,BLCA** |
| Cox6b2 | 333182 | COX6B2 | 125965 | -8.78 | **PAAD** |
| Crispld2 | 78892 | CRISPLD2 | 83716 | -3.89 | **KIRC,KIRP** |
| Ctsk | 13038 | CTSK | 1513 | -4.94 | **KIRC** |
| Diablo | 66593 | DIABLO | 56616 | -1.34 | **KIRC,KIRP,KICH** |
| Dkk3 | 50781 | DKK3 | 27122 | -3.65 | **KIRC,KIRP,KICH** |
| Eln | 13717 | ELN | 2006 | -5.23 | **THCA** |
| Emilin1 | 100952 | EMILIN1 | 11117 | -3.98 | **KICH** |
| Eng | 13805 | ENG | 2022 | -2.42 | **KICH,LIHC,HNSC** |
| Enoph1 | 67870 | ENOPH1 | 58478 | -1.47 | **LIHC** |
| Fgfr3 | 14184 | FGFR3 | 2261 | -1.99 | **UCEC** |
| Fhit | 14198 | FHIT | 2272 | -2.81 | **KIRP** |
| Fhod1 | 234686 | FHOD1 | 29109 | -1.99 | **KIRC,KIRP,KICH,CESC** |
| Fstl1 | 14314 | FSTL1 | 11167 | -3.91 | **KICH** |
| Fzd1 | 14362 | FZD1 | 8321 | -2.50 | **KIRC,KIRP,KICH** |
| Gas1 | 14451 | GAS1 | 2619 | -5.14 | **THCA,KIRC,KIRP,KICH,CAD,STAD** |
| Gdf6 | 242316 | GDF6 | 392255 | -6.70 | **KIRC,KIRP,KICH** |
| Gpn1 | 74254 | GPN1 | 11321 | -1.41 | **LIHC,UCEC** |
| Gprc5b | 64297 | GPRC5B | 51704 | -3.82 | **COAD,KIRC,KIRP,KICH** |
| Gpx3 | 14778 | GPX3 | 2878 | -4.76 | **STAD** |
| Gsta1 | 14857 | GSTA1 | 2938 | 7.38 | **KIRC,KICH,LUSC** |
| Hrg | 94175 | HRG | 3273 | 2.88 | **LIHC** |
| Hspb7 | 29818 | HSPB7 | 27129 | -9.53 | **BLCA,HSC** |
| Kcnq1 | 16535 | KCNQ1 | 3784 | -4.95 | **STAD** |
| Klf12 | 16597 | KLF12 | 11278 | 1.39 | **UCEC** |
| Lama4 | 16775 | LAMA4 | 3910 | -3.80 | **KIRP, KICH** |
| Lamb1 | 16777 | LAMB1 | 3912 | -3.47 | **LIHC** |
| Lbh | 77889 | LBH | 81606 | -4.03 | **STAD** |
| Loxl1 | 16949 | LOXL1 | 4016 | -4.73 | **GBM** |
| Ltf | 17002 | LTF | 4057 | -14.11 | **KIRC,KIRP** |
| Lum | 17022 | LUM | 4060 | -5.59 | **KICH** |
| Me2 | 107029 | ME2 | 4200 | -3.99 | **LIHC** |
| Mgp | 17313 | MGP | 4256 | -7.46 | **KICH** |
| Mmp9 | 17395 | MMP9 | 4318 | -7.43 | **KIRC,LIHC, UCEC** |
| Mrc2 | 17534 | MRC2 | 9902 | -3.95 | **KIRC,OVCA** |
| Mrpl33 | 66845 | MRPL33 | 9553 | -1.52 | **LIHC,HNSC** |
| Muc13 | 17063 | MUC13 | 56667 | -4.52 | **KIRC** |
| Mxra8 | 74761 | MXRA8 | 54587 | -4.28 | **KICH,BLCA,STAD** |
| Ncoa6 | 56406 | NCOA6 | 23054 | 1.50 | **KIRC,LIHC** |
| Nid2 | 18074 | NID2 | 22795 | -2.31 | **STAD, BLCA** |
| Olfml3 | 99543 | OLFML3 | 56944 | -3.83 | **KIRC,KIRP,KICH** |
| Pdgfra | 18595 | PDGFRA | 5156 | -4.07 | **KIRC,KIRP,HNSC** |
| Ppic | 19038 | PPIC | 5480 | -3.71 | **LIHC** |
| Prex2 | 109294 | PREX2 | 80243 | -2.05 | **KICH** |
| Procr | 19124 | PROCR | 10544 | -3.71 | **KIRP** |
| Prrx1 | 18933 | PRRX1 | 5396 | -9.98 | **KIRC,KIRP,KICH** |
| Prss23 | 76453 | PRSS23 | 11098 | -4.78 | **HNSC,KIRC,KIRP,PAAD** |
| Ptgis | 19223 | PTGIS | 5740 | -5.35 | **Renal,BLCA** |
| Rab11fip3 | 215445 | RAB11FIP3 | 9727 | 1.41 | **PAAD, LIHC,UCEC,KIRC, KIHC** |
| Rab25 | 53868 | RAB25 | 57111 | -15.28 | **KIRC** |
| Rgs3 | 50780 | RGS3 | 5998 | -2.67 | **LIHC,BRCA** |
| Robo1 | 19876 | ROBO1 | 6091 | -8.11 | **LIHC** |
| S100a8 | 20201 | S100A8 | 6279 | -20.82 | **KICH** |
| S100a9 | 20202 | S100A9 | 6280 | -18.31 | **LIHC,KIRC** |
| Scarf2 | 224024 | SCARF2 | 91179 | -4.29 | **KIRC,KIRP,BLCA** |
| Serpinh1 | 12406 | SERPINH1 | 871 | -3.17 | **KIRC,KIRP,HNSC,CESC** |
| Setbp1 | 240427 | SETBP1 | 26040 | -4.03 | **KICH** |
| Slit3 | 20564 | SLIT3 | 6586 | -7.77 | **KIRC,KIRP** |
| Sncg | 20618 | SNCG | 6623 | -7.01 | **KIRC,KIRP,KICH,STAD** |
| Srxn1 | 76650 | SRXN1 | 140809 | 2.23 | **LIHC** |
| Stk39 | 53416 | STK39 | 27347 | -4.16 | **LIHC,BRCA** |
| Tagln | 21345 | TAGLN | 6876 | -7.24 | **KICH** |
| Tap1 | 21354 | TAP1 | 6890 | 1.60 | **KIRP,OVSC,COAD** |
| Tfpi | 21788 | TFPI | 7035 | -3.04 | **CESC** |
| Tgfb1i1 | 21804 | TGFB1I1 | 7041 | -4.22 | **KIRP,BLCA** |
| Timp3 | 21859 | TIMP3 | 7078 | -3.45 | **OVCA,KICH** |
| Tm4sf1 | 17112 | TM4SF1 | 4071 | -3.45 | **BLCA,KICH,PAAD** |
| Tmem204 | 407831 | TMEM204 | 79652 | -2.86 | **LIHC,SKCM** |

**Supplementary Table 4. Functional categorization of the 149 transcripts shown in Fig. 4E.** Using Ingenuity Pathway Analysis profiling these could be grouped into major pathways that included mitochondrial or ETC function, ribosome/EIF2 pathways and remodeling of epithelial cell adherens junctions.

| **NCBI  Gene Symbol** | **NCBI  Gene ID** | **Fold Change (Δ(90)+ YAPS127A DDDA 3 wks vs Ctrl Diet)** | **Fold Change (Δ(90)+ YAP^S127A^ DDDA >20 wks vs Ctrl Diet)** | **Fold Change (Δ(90)+ YAP^S127A^ DDDA >20 wks vs DDDA 3 wks)** | **Ingenuity Canonical Pathways** |
| --- | --- | --- | --- | --- | --- |
| Abcc3 | 76408 | 2.42 | 4.24 | 1.75 | EIF2 Signaling,LPS/IL-1 Mediated Inhibition of RXR Function, |
| Fmo4 | 226564 | 2.30 | 2.84 | 1.23 | EIF2 Signaling,LPS/IL-1 Mediated Inhibition of RXR Function, |
| Myh14 | 71960 | 2.08 | 2.26 | 1.09 | ILK Signaling, |
| Rsu1 | 20163 | 1.69 | 1.96 | 1.16 | ILK Signaling, |
| Alpl | 11647 | 4.38 | 2.93 | -1.49 | Phosphatidylglycerol Biosynthesis II (Non-plastidic) |
| Rarres2 | 71660 | 3.13 | 2.79 | -1.12 | Inosine-5'-phosphate Biosynthesis II, |
| Map2k4 | 26398 | 1.61 | 1.89 | 1.17 | Mitochondrial Dysfunction,Coronavirus Pathogenesis Pathway,ILK Signaling,EIF2 Signaling,LPS/IL-1 Mediated Inhibition of RXR Function, |
| Casp3 | 12367 | 1.51 | 1.59 | 1.05 | Mitochondrial Dysfunction,Coronavirus Pathogenesis Pathway,ILK Signaling,Inosine-5'-phosphate Biosynthesis II, |
| Maoa | 17161 | 2.50 | 3.56 | 1.42 | Mitochondrial Dysfunction,EIF2 Signaling,LPS/IL-1 Mediated Inhibition of RXR Function, |
| Ndufa3 | 66091 | -2.06 | -2.31 | -1.12 | Mitochondrial Dysfunction,Oxidative Phosphorylation,Sirtuin Signaling Pathway, |
| Ndufb11 | 104130 | -1.76 | -1.66 | 1.06 | Mitochondrial Dysfunction,Oxidative Phosphorylation,Sirtuin Signaling Pathway, |
| Ndufb2 | 68198 | -1.86 | -2.32 | -1.24 | Mitochondrial Dysfunction,Oxidative Phosphorylation,Sirtuin Signaling Pathway, |
| Ndufs6 | 407785 | -2.24 | -2.22 | 1.01 | Mitochondrial Dysfunction,Oxidative Phosphorylation,Sirtuin Signaling Pathway, |
| Atp5e | 67126 | -1.79 | -1.90 | -1.06 | Mitochondrial Dysfunction,Sirtuin Signaling Pathway, |
| Timm13 | 30055 | -1.70 | -2.16 | -1.27 | Oxidative Phosphorylation, |
| Tomm7 | 66169 | -1.78 | -2.18 | -1.22 | Oxidative Phosphorylation, |
| Hif1a | 15251 | 1.75 | 1.88 | 1.07 | HIF1α Signaling,Oxidative Phosphorylation,ILK Signaling,Inhibition of Angiogenesis by TSP1,Inosine-5'-phosphate Biosynthesis II, |
| Rpl22l1 | 68028 | -2.40 | -2.63 | -1.10 | EIF2 Signaling |
| Rpl35a | 57808 | -2.80 | -2.24 | 1.25 | EIF2 Signaling |
| Rps18 | 20084 | -1.94 | -1.69 | 1.15 | EIF2 Signaling |
| Rps28 | 54127 | -1.93 | -1.65 | 1.17 | EIF2 Signaling |
| Rps29 | 20090 | -1.99 | -2.35 | -1.18 | EIF2 Signaling |
| Serpine1 | 18787 | 8.17 | 4.85 | -1.68 | HIF1α Signaling |
| 1110001J03Rik | 66117 | -2.56 | -2.46 | 1.04 |  |
| 2410015M20Rik | 224904 | -2.03 | -2.04 | -1.00 |  |
| 2510009E07Rik | 72190 | 1.98 | 1.54 | -1.29 |  |
| 4931406H21Rik | 77592 | -11.88 | -3.22 | 3.68 |  |
| A330023F24Rik | 320977 | -3.18 | -4.10 | -1.29 |  |
| A330049N07Rik | 327768 | -6.18 | -7.29 | -1.18 |  |
| Abca5 | 217265 | 1.94 | 2.37 | 1.22 |  |
| Acp5 | 11433 | 1.78 | 1.79 | 1.01 |  |
| Agfg1 | 15463 | 1.72 | 1.86 | 1.09 |  |
| Akr1b3 | 11677 | 1.79 | 1.35 | -1.33 |  |
| Aldoa | 11674 | 2.27 | 1.86 | -1.22 |  |
| Alg14 | 66789 | 1.74 | 1.69 | -1.03 |  |
| Anapc13 | 69010 | -2.13 | -2.00 | 1.06 |  |
| Ankrd12 | 106585 | -1.88 | -1.53 | 1.22 |  |
| Arglu1 | 234023 | -1.68 | -1.86 | -1.11 |  |
| Arhgap18 | 73910 | 1.66 | 1.49 | -1.11 |  |
| Asph | 65973 | 1.50 | 1.48 | -1.01 |  |
| Atic | 108147 | 1.70 | 1.96 | 1.16 |  |
| Atp5k | 11958 | -2.61 | -3.35 | -1.28 |  |
| Atp6ap1 | 54411 | 1.65 | 1.62 | -1.02 |  |
| AW549542 | 100993 | -4.49 | -4.29 | 1.05 |  |
| B3galnt1 | 26879 | 2.82 | 2.93 | 1.04 |  |
| BC005561 | 100042165 | -2.37 | -2.10 | 1.13 |  |
| Bhlhe40 | 20893 | 2.25 | 1.92 | -1.17 |  |
| Bri3 | 55950 | 1.67 | 1.46 | -1.15 |  |
| Cdc42ep5 | 58804 | 2.73 | 2.59 | -1.06 |  |
| Cdh13 | 12554 | 17.50 | 5.42 | -3.23 |  |
| Cep170 | 545389 | 1.69 | 1.99 | 1.18 |  |
| Col12a1 | 12816 | 5.61 | 2.38 | -2.36 |  |
| Commd10 | 69456 | 1.64 | 1.55 | -1.06 |  |
| Cox7c | 12867 | -1.85 | -1.82 | 1.02 |  |
| Cse1l | 110750 | 1.71 | 1.63 | -1.05 |  |
| Ctr9 | 22083 | 1.73 | 1.56 | -1.10 |  |
| Cxx1a | 66158 | 2.19 | 1.81 | -1.21 |  |
| Dcakd | 68087 | 1.62 | 1.57 | -1.03 |  |
| Dpm3 | 68563 | -1.72 | -1.56 | 1.10 |  |
| Drap1 | 66556 | -1.68 | -1.68 | -1.00 |  |
| Egln3 | 112407 | 3.76 | 2.63 | -1.43 |  |
| Elovl1 | 54325 | 1.65 | 1.55 | -1.06 |  |
| Elp2 | 58523 | 1.54 | 1.55 | 1.01 |  |
| Fam193b | 212483 | -1.79 | -2.45 | -1.37 |  |
| Fam196a | 627214 | -20.06 | -5.34 | 3.75 |  |
| Fam20a | 208659 | 1.78 | 2.12 | 1.19 |  |
| Fam25c | 69134 | -4.35 | -3.50 | 1.24 |  |
| Fam96b | 68523 | -1.68 | -1.85 | -1.10 |  |
| Gcnt2 | 14538 | 1.75 | 1.84 | 1.05 |  |
| Gm10277 | 791303 | -5.77 | -4.51 | 1.28 |  |
| Gm20752 | 624759 | -5.23 | -5.93 | -1.13 |  |
| Gm7120 | 633640 | 1.94 | 1.69 | -1.15 |  |
| Gpx3 | 14778 | 10.82 | 2.27 | -4.76 |  |
| Gstm5 | 14866 | 1.64 | 1.53 | -1.08 |  |
| Hapln4 | 330790 | 2.77 | 3.94 | 1.42 |  |
| Hspe1 | 15528 | -1.84 | -1.49 | 1.24 |  |
| Hypk | 67693 | -1.64 | -1.43 | 1.14 |  |
| Idnk | 75731 | -1.65 | -1.47 | 1.13 |  |
| Igfbp6 | 16012 | 10.53 | 5.39 | -1.95 |  |
| Inhba | 16323 | -3.21 | -2.90 | 1.11 |  |
| Jph3 | 57340 | -22.24 | -51.19 | -2.30 |  |
| Kcnq1ot1 | 63830 | -6.90 | -2.98 | 2.31 |  |
| Kif5a | 16572 | -3.67 | -3.32 | 1.11 |  |
| Krt10 | 16661 | -2.99 | -2.37 | 1.26 |  |
| Lars2 | 102436 | 2.30 | 2.86 | 1.24 |  |
| Lclat1 | 225010 | 1.61 | 1.69 | 1.05 |  |
| Ldlrad3 | 241576 | 2.20 | 2.15 | -1.02 |  |
| Leap2 | 259301 | -3.52 | -3.59 | -1.02 |  |
| Lsm7 | 66094 | -2.01 | -2.33 | -1.16 |  |
| Luc7l3 | 67684 | -1.82 | -1.82 | 1.00 |  |
| Malat1 | 72289 | -2.78 | -1.55 | 1.79 |  |
| Manea | 242362 | 1.73 | 1.53 | -1.13 |  |
| Mapre2 | 212307 | 1.63 | 1.44 | -1.13 |  |
| Mapre3 | 100732 | 1.86 | 1.65 | -1.13 |  |
| Mif | 17319 | 1.89 | 1.65 | -1.15 |  |
| Minos1 | 433771 | -1.64 | -2.11 | -1.29 |  |
| Muc6 | 353328 | -17.24 | -11.24 | 1.53 |  |
| Mup10 | 100039008 | -28.34 | -10.00 | 2.83 |  |
| Myeov2 | 66915 | -1.97 | -2.02 | -1.03 |  |
| Myo7b | 17922 | 13.77 | 5.54 | -2.49 |  |
| Ndufs5 | 595136 | -1.92 | -1.94 | -1.01 |  |
| Nme3 | 79059 | -1.79 | -1.69 | 1.06 |  |
| Nostrin | 329416 | 1.87 | 2.08 | 1.11 |  |
| Nrp | 654309 | -17.72 | -19.63 | -1.11 |  |
| Nt5dc1 | 319638 | 1.77 | 1.70 | -1.04 |  |
| Ntm | 235106 | -4.05 | -3.59 | 1.13 |  |
| Pgd | 110208 | 1.74 | 1.79 | 1.02 |  |
| Phpt1 | 75454 | -2.16 | -1.63 | 1.32 |  |
| Picalm | 233489 | 1.73 | 1.65 | -1.05 |  |
| Pla1a | 85031 | 2.01 | 1.85 | -1.08 |  |
| Ppig | 228005 | -1.61 | -1.26 | 1.28 |  |
| Ppme1 | 72590 | 1.60 | 1.56 | -1.02 |  |
| Ppt2 | 54397 | 1.56 | 1.62 | 1.04 |  |
| Prune | 229589 | 1.90 | 2.36 | 1.24 |  |
| Raver1 | 71766 | 1.67 | 1.54 | -1.09 |  |
| Rbpms | 19663 | 1.71 | 1.95 | 1.14 |  |
| Rho | 212541 | 35.02 | 2.45 | -14.28 |  |
| Robo1 | 19876 | 3.80 | -2.13 | -8.11 |  |
| Romo1 | 67067 | -1.85 | -1.59 | 1.16 |  |
| Scyl2 | 213326 | 1.62 | 1.70 | 1.05 |  |
| Sdcbp | 53378 | 1.68 | 1.27 | -1.32 |  |
| Sec14l3 | 380683 | 255.57 | 2.97 | -86.00 |  |
| Sf3a2 | 20222 | 1.92 | 1.67 | -1.15 |  |
| Sf3b4 | 107701 | 1.75 | 1.66 | -1.06 |  |
| Sgk1 | 20393 | 5.14 | 3.62 | -1.42 |  |
| Sik1 | 17691 | 2.36 | 1.38 | -1.71 |  |
| Slc35g1 | 240660 | 1.87 | 1.93 | 1.03 |  |
| Slco1a6 | 28254 | 9.59 | 2.81 | -3.42 |  |
| Snhg20 | 76972 | -2.40 | -3.83 | -1.59 |  |
| Snrpc | 20630 | 1.60 | 1.59 | -1.00 |  |
| Stom | 13830 | 2.09 | 2.77 | 1.33 |  |
| Sub1 | 20024 | -1.52 | -1.55 | -1.02 |  |
| Tbca | 21371 | -1.81 | -1.80 | 1.00 |  |
| Tmem209 | 72649 | 1.68 | 1.84 | 1.09 |  |
| Tmem258 | 69038 | -1.86 | -1.98 | -1.07 |  |
| Tmsb10 | 19240 | 4.30 | 3.08 | -1.40 |  |
| Tpp1 | 12751 | 1.60 | 1.69 | 1.05 |  |
| Tpp2 | 22019 | 1.59 | 1.85 | 1.17 |  |
| Ube2d2a | 56550 | 1.53 | 1.46 | -1.05 |  |
| Ubl7 | 69459 | 1.62 | 1.76 | 1.09 |  |
| Ubqln2 | 54609 | 1.94 | 2.65 | 1.37 |  |
| Uqcc2 | 67267 | -1.72 | -1.99 | -1.16 |  |
| Vamp5 | 53620 | -2.13 | -1.92 | 1.11 |  |
| Vat1 | 26949 | 2.64 | 2.31 | -1.14 |  |
| Zfas1 | 68949 | -2.92 | -3.87 | -1.32 |  |
| Zfhx3 | 11906 | -2.30 | -1.61 | 1.43 |  |
| Zfp872 | 619310 | -5.68 | -4.35 | 1.31 |  |
| Zfp949 | 71640 | -1.65 | -1.55 | 1.06 |  |
| Zyx | 22793 | 2.10 | 1.80 | -1.16 |  |

**Supplementary Table 5. Putative functions of the 12 up-regulated transcripts depicted in Fig. 4E that most strikingly distinguish >20 wk DDDA tumors from control diet tumors and 3 wk DDDA tumors.** The function of each transcript’s encoded protein was obtained from the GeneCards Database (https://www.genecards.org/).

| **NCBI  Gene Symbol** | **NCBI  Gene ID** | **Fold Change (Δ(90)+ YAPS127A DDDA >20 wks vs DDDA 3 wks)** | **Putative functions** |
| --- | --- | --- | --- |
| Akr1b3 | 11677 | -1.33 | Aldo-keto reductase family |
| Cdh13 | 12554 | -3.23 | calcium-dependent cell adhesion proteins; endothelial survival |
| Col12a1 | 12816 | -2.36 | the FACIT (fibril-associated collagens with interrupted triple helices) collagen family |
| Gpx3 | 14778 | -4.76 | Glutathione peroxidase; secreted anti-oxidant |
| Igfbp6 | 16012 | -1.95 | Insulin-like growth factor binding protein; alters IGF cell surface binding |
| Myo7b | 17922 | -2.49 | involved in linking protocadherins to the actin cytoskeleton; functions in endocytosis |
| Rho | 212541 | -14.28 | Rhodopsin |
| Robo1 | 19876 | -8.11 | Roundabout guidance receptor; cell guidance |
| Sdcbp | 53378 | -1.32 | links syndecan-mediated signaling to the cytoskeleton; cell adhesion |
| Sec14l3 | 380683 | -86 | Phosphatidylinositol transfer protein; role in biogenesis of Golgi-derived transport vesicles |
| Sik1 | 17691 | -1.71 | Adenosine monophosphate-activated kinase (AMPK) subfamily; putative tumor suppressor |
| Slco1a6 | 28254 | -3.42 | Solute carrier organic anion transporter family; bile acid transport |
